# The storage capacity of a directed graph and nodewise autonomous, ubiquitous learning

**DOI:** 10.3389/fncom.2023.1254355

**Published:** 2023-10-19

**Authors:** Hui Wei, Fushun Li

**Affiliations:** Laboratory of Algorithms for Cognitive Models, School of Computer Science, Shanghai Key Laboratory of Data Science, Fudan University, Shanghai, China

**Keywords:** directed graph, storage, parallel distributed learning, memory model, associative memory

## Abstract

The brain, an exceedingly intricate information processing system, poses a constant challenge to memory research, particularly in comprehending how it encodes, stores, and retrieves information. Cognitive psychology studies memory mechanism from behavioral experiment level and fMRI level, and neurobiology studies memory mechanism from anatomy and electrophysiology level. Current research findings are insufficient to provide a comprehensive, detailed explanation of memory processes within the brain. Numerous unknown details must be addressed to establish a complete information processing mechanism connecting micro molecular cellular levels with macro cognitive behavioral levels. Key issues include characterizing and distributing content within biological neural networks, coexisting information with varying content, and sharing limited resources and storage capacity. Compared with the hard disk of computer mass storage, it is very clear from the polarity of magnetic particles in the bottom layer, the division of tracks and sectors in the middle layer, to the directory tree and file management system in the high layer, but the understanding of memory is not sufficient. Biological neural networks are abstracted as directed graphs, and the encoding, storage, and retrieval of information within directed graphs at the cellular level are explored. A memory computational model based on active directed graphs and node-adaptive learning is proposed. First, based on neuronal local perspectives, autonomous initiative, limited resource competition, and other neurobiological characteristics, a resource-based adaptive learning algorithm for directed graph nodes is designed. To minimize resource consumption of memory content in directed graphs, two resource-occupancy optimization strategies—lateral inhibition and path pruning—are proposed. Second, this paper introduces a novel memory mechanism grounded in graph theory, which considers connected subgraphs as the physical manifestation of memory content in directed graphs. The encoding, storage, consolidation, and retrieval of the brain's memory system correspond to specific operations such as forming subgraphs, accommodating multiple subgraphs, strengthening connections and connectivity of subgraphs, and activating subgraphs. Lastly, a series of experiments were designed to simulate cognitive processes and evaluate the performance of the directed graph model. Experimental results reveal that the proposed adaptive connectivity learning algorithm for directed graphs in this paper possesses the following four features: (1) Demonstrating distributed, self-organizing, and self-adaptive properties, the algorithm achieves global-level functions through local node interactions; (2) Enabling incremental storage and supporting continuous learning capabilities; (3) Displaying stable memory performance, it surpasses the Hopfield network in memory accuracy, capacity, and diversity, as demonstrated in experimental comparisons. Moreover, it maintains high memory performance with large-scale datasets; (4) Exhibiting a degree of generalization ability, the algorithm's macroscopic performance remains unaffected by the topological structure of the directed graph. Large-scale, decentralized, and node-autonomous directed graphs are suitable simulation methods. Examining storage problems within directed graphs can reveal the essence of phenomena and uncover fundamental storage rules hidden within complex neuronal mechanisms, such as synaptic plasticity, ion channels, neurotransmitters, and electrochemical activities.

## 1. Introduction

In computer science, a graph serves as a data structure for modeling elements from a specific set and their pairwise relationships. A graph comprises a set of nodes and a set of edges connecting node pairs (Biggs et al., 1986). As a classical data structure, graphs offer an abstract representation of system components and their interactions when depicting natural and engineering systems. Consequently, graph theory is extensively applied in studying multiple relationships and process dynamics across disciplines such as computer science, economics, biology, chemistry, and social sciences.

Directed graphs can characterize systems in various domains, including road traffic, social networks, ecological networks, neural systems, and financial networks. Traditional directed graph models tend to be static, focusing on the structure of directed graphs, i.e., the static properties of elements and their relationships within a set. Static directed graphs, lacking dynamic behavior, primarily function as data structures for recording data from a global perspective. However, most applications involve dynamic interactions between system elements, necessitating the construction of dynamic directed graph models. These models can be divided into constant-topology dynamic directed graphs and variable-topology dynamic directed graphs, depending on whether the graph topology changes. Constant-topology dynamic directed graph models encompass flow models (Ahuja et al., 1993), graph random walks and diffusion models (Blanchard and Volchenkov, 2011; Riascos et al., 2020). Variable-topology dynamic directed graph models include growth models (Barabási and Albert, 1999) and random evolution models (Watts and Strogatz, 1998).

Multi-agent systems are often characterized by graph structures, which facilitate the emergence of global behavior through local interactions such as cluster collaboration or holistic optimization (Chen et al., 2019). Dynamic directed graphs, based on multi-agent systems, demonstrate distributed computing and self-organization. This category of dynamic directed graphs exhibits two primary characteristics: (1) Nodes in a directed graph have autonomous computational capabilities, rather than simply functioning as numerical representations. The internal properties of each node, along with upstream and downstream node interactions, vary and are computed independently. (2) In the directed graph, there is no centralized decision-maker or omniscient perspective; each node can only adapt its behavior based on local neighborhood information. Numerous examples of dynamic directed graph models based on multi-agent systems exist in nature, including biological neural networks, ant colonies, and bee colonies.

Directed graphs may consist of a vast number of nodes and edges, forming large, distinct connected subgraphs. If a connected subgraph is regarded as a state representation, a directed graph can potentially offer a considerable resource based on the diversity of combinations. A direct application involves utilizing such dynamic directed graphs, which are distributed, self-organizing, adaptive, easily scalable, and loss-resistant, for implementing associative memory. A connected subgraph represents the physical realization of memory content in a directed graph, potentially providing substantial storage capacity. A directed graph can contain numerous connected subgraphs corresponding to different memory contents. The self-organized incremental learning of directed graphs enables the distinction, compatibility, minimal interference, and efficient use of limited node resources among connected subgraphs. The challenge in this research is ensuring collaboration and optimization of all nodes autonomously and independently without a centralized perspective. Such a directed graph transforms from a static recorder or passive visitor awaiting traversal by a search algorithm into a dynamic network of capable nodes.

In this paper, we integrate concepts from neurobiology, graph theory, multi-agent systems, parallel distributed processing, and ubiquitous learning to construct directed graphs that mirror the connectivity properties of biological cortical neural networks. We design node adaptive connectivity learning algorithms based on limited resource competition to investigate the storage capacity of an active directed graph from a graph theory perspective.

The main contributions of this work are summarized as follows.

(1) we design directed graphs based on biological cortical neural networks, with nodes having autonomy and limited resources, and the node adaptive connectivity learning algorithm achieves associative memory through local information, aligning more closely with neurobiology.
(2) we apply active directed graphs to associative memory and propose a novel memory mechanism based on graph theory, considering connected subgraphs as directed graph resources related to memory capacity.
(3) The directed graph model demonstrates consistent memory performance, outperforming the Hopfield network in terms of memory accuracy, capacity, and diversity, as evidenced by experimental comparisons. It also maintains strong memory performance when handling large-scale datasets.
(4) The directed graph model facilitates incremental storage and facilitates continuous learning capabilities.

## 2. Related work

Graphs represent a significant data structure in various fields, such as computer science, neuroscience, and sociology, leading to graph theory becoming a popular research area for many scholars. Graph theory has demonstrated unique advantages in domains such as big data analysis (Yıldırım et al., 2021), brain networks (Bullmore and Sporns, 2009; He and Evans, 2010; Zhao et al., 2019; Sporns, 2022), social networks (Trolliet et al., 2022), clustering (Malliaros and Vazirgiannis, 2013; Gao et al., 2022), security (He et al., 2019; Zhu et al., 2019), and robotics (Cheng et al., 2019; Lyu et al., 2021). In studies related to associative memory, graph theory is primarily employed to analyze the impact of network topology on associative memory performance. Kaviani and Sohn (2021) examined associative memory performance in different networks, including random complex networks, small-world networks, scale-free networks, and regular networks. Berend et al. (2014) analyzed the influence of network topology on the stability of Hopfield networks. The aforementioned research on graph theory-based associative memory primarily focuses on static networks and explores the impact of network topology on associative memory.

Associative memory network models store memory information in the weights of neuronal networks, with the dynamic evolution of the network representing associative memory. Associative memory is classified into self-associative memory and hetero-associative memory. Self-associative memory outputs a memory sample based on partial input memory sample information, while hetero-associative memory retrieves other memory samples related to the input memory sample. Classical associative memory models, such as Hopfield network (Hopfield, 1982) and BAM network (Kosko, 1988), leverage neural dynamics for information storage and retrieval. The Hopfield network is a single-layer feedback neural network with interconnected neurons that function as both input and output units. The BAM network is a two-layer bidirectional neural network enabling hetero-associative memory. Energy functions are defined in both Hopfield and BAM networks, where the network's stable states correspond to local energy minima. These stable states, called attractors, provide the basis for distributed memory storage of information.

Hopfield network, BAM network, and their improved models (Knoblauch and Palm, 2019; Marullo and Agliari, 2020; Ladwani and Ramasubramanian, 2021; Li et al., 2021; Sun et al., 2021), although used as computational models for memory implementation, do not satisfy neurobiological constraints on the connectivity properties of biological cortical networks. The high regularity of connection methods, the preset weight matrix, and the limited capacity of these models preclude considering them as explorations of the internal memory realization mechanisms. At the neuronal network level, the design of node connections in these network models does not correspond to biological reality. The probability of establishing connections between cortical regions or neurons decreases rapidly with increasing distance in primate cortical and Caenorhabditis elegans neuronal networks.

According to neurobiology, associative memory models should be characterized by local connections rather than extensive connections. Consequently, many studies have applied small-world networks and random networks to associative memory. Duan et al. (2016) proposed a novel associative memory model based on small-world networks and memristors, exhibiting similar performance to the fully connected Hopfield network in sparse networks. Löwe and Vermet (2015) demonstrated that associative memory models based on small-world networks possess comparable memory performance to random networks while using fewer network resources (Bohland and Minai, 2001). Löwe et al. developed a concept of storage capacity associated with graph topology and analyzed the storage capacity of Hopfield networks based on random graphs. Although these models adhere to the neurobiological constraints of local connectivity, their nodes are simple numerical representations lacking autonomous computation abilities.

Inspired by brain networks, many scholars have utilized different neurobiological features to construct associative memory models, respectively. Inspired by the chunking mechanism of the brain, Huang et al. (2019) proposed an associative memory model based on the chunking mechanism. The model has an associative memory recall module (AMR) and a learning module (KID), respectively. The KID model is used to learn associative knowledge, while the AMR continuously searches for associations between knowledge units and uses a merging mechanism to merge the relevant units. Tyulmankov et al. (2021) proposed a key-value-based associative memory model, KVMN (Key-Value Memory Network), which uses biologically plausible three-element combinations to store inputs. Salvatori et al. (2021) proposed an associative memory model based on predictive coding, which imitates the behavior of the hippocampus as a memory indexing and generating model. These associative memory models described above need to be manipulated by an algorithm with a global perspective. However, biological neurons themselves are complex information processing units, and such a design ignores the micro-level complexity of the molecular mechanisms and subcellular structures behind biological neurons, which allows each unit to operate independently, learn autonomously, and adjust based on local information, without the need for a supercommand to command from above.

In neurobiology, current memory models based on dynamic directed graphs focus more on changes in the topology of the directed graph than on the dynamic behavior of the directed graph nodes themselves. Millán et al. (2021) propose an adaptive neural network model that reproduces the temporal distribution of synaptic density observed in the brain. In that study it was shown that intermediate synaptic densities provide optimal developmental pathways with minimal energy, providing a viable design strategy for building neural networks with specific information processing capabilities. In another research work by Millán et al. (2019) it was explored how evolutionary mechanisms of brain structure affect memory storage processes. The interaction of brain regions at the network level may provide the necessary infrastructure for the development of cognitive processes. The work of Woodburn et al. (2021) describes the maturation of network separation and integration in the child's brain, suggesting that a specific trajectory of maturation of brain networks contributes to cognitive outcomes after growth. The research work described above has focused on studying the structural and functional connectivity of neurons or brain regions and has not examined the information processing mechanisms of memory at the scale of neuronal networks.

## 3. Design of directed graphs based on real biological neural networks

In this section, a limited resource competition mechanism and a directed graph generation strategy are designed based on neurobiology, and the implementation of memory content in dynamic directed graphs is illustrated from a graph-theoretic perspective.

### 3.1. Directed graph path resources

The competition for limited resources is crucial in shaping individual neuron morphology and establishing interneuron connections. This paper proposes a limited resource competition mechanism in active directed graphs, inspired by this neurobiological feature. Axonal migration to the target is facilitated by neurotrophic factors during axonal growth (Tessier-Lavigne and Goodman, 1996; Alsina et al., 2001). In the mature nervous system, a finite number of neurotrophic factors play a vital role in regulating synaptic function, synaptic plasticity, and neural network remodeling (Huang and Reichardt, 2001; Jeanneteau et al., 2020). The competition mechanism involving neurotrophic factors is characterized by a restricted supply to target neurons and dynamically adjustable topological connections between neurons. The concept of limited resources is evident not only in the competition among different neuronal axons for the target neuron but also in the tubulin dynamics revealing competition between neurites of the same neuron (Van Ooyen, 2005; Hjorth et al., 2014). Moreover, limited resource competition mechanisms have been demonstrated for calmodulin (Okamoto and Ichikawa, 2000), PSD (surface area of the postsynaptic density) (Bourne and Harris, 2011), AMPAR (Triesch et al., 2018), and synaptic space (Takeo et al., 2021).

These studies highlight the significance of limited resource competition mechanisms in neuronal signal processing. In the directed graph model proposed here, the total amount of postsynaptic resources for a single neuron is maintained constant, and the signal strength of the input neuron is related to the postsynaptic resources it competes for, thus abstracting biological neurons as nodes with a constant total contact area in the directed graph.

### 3.2. Directed graph generation strategy

According to neurobiology, the topological and physical distances between neurons in brain neural networks are typically intricately related. Connections between spatially proximate neurons or brain regions are relatively likely, while connections between spatially distant neurons or regions are less probable (Averbeck and Seo, 2008). The reasons behind the probabilistic distance dependence of connectivity in brain neural networks have been extensively debated. Borisyuk et al. (2008) suggested that predetermined deterministic rules in genetic instructions play a dominant role in neural growth, while Braitenberg and Schüz (2013) posited that nerve growth is fundamentally stochastic, with connection specificity correlating with the overlap of specific sets of neurons. This paper designs the directed graph structure based on the latter viewpoint. The directed graph model's primary feature is that its nodes form finite and random connections within the local neighborhood, and the connection probability varies with the distance between nodes.

Neural networks in the biological cerebral cortex exhibit variations among individuals and localities. These networks may only demonstrate statistical consistency without identical details. Consequently, a directed graph generation strategy is essential for batch-building large-scale, statistically similar directed graphs for performance testing. The directed graph features five characteristics: (1) The out-degree and in-degree of each node may vary. (2) The connection probability between nodes is distance-dependent, favoring short-range connections over long-range ones. (3) The contact area of inter-nodal connections corresponds to the synaptic efficacy of neurons, and each node's contact area is randomly generated within a predetermined range. (4) Each node accumulates input values from its upstream nodes, which positively correlate with the occupied contact area. (5) The activation threshold of each node is randomly generated within a specified range. When a node's cumulative input value exceeds the threshold, the node activates, accesses its downstream nodes, and clears the cumulative input value.

**Figure 1:**
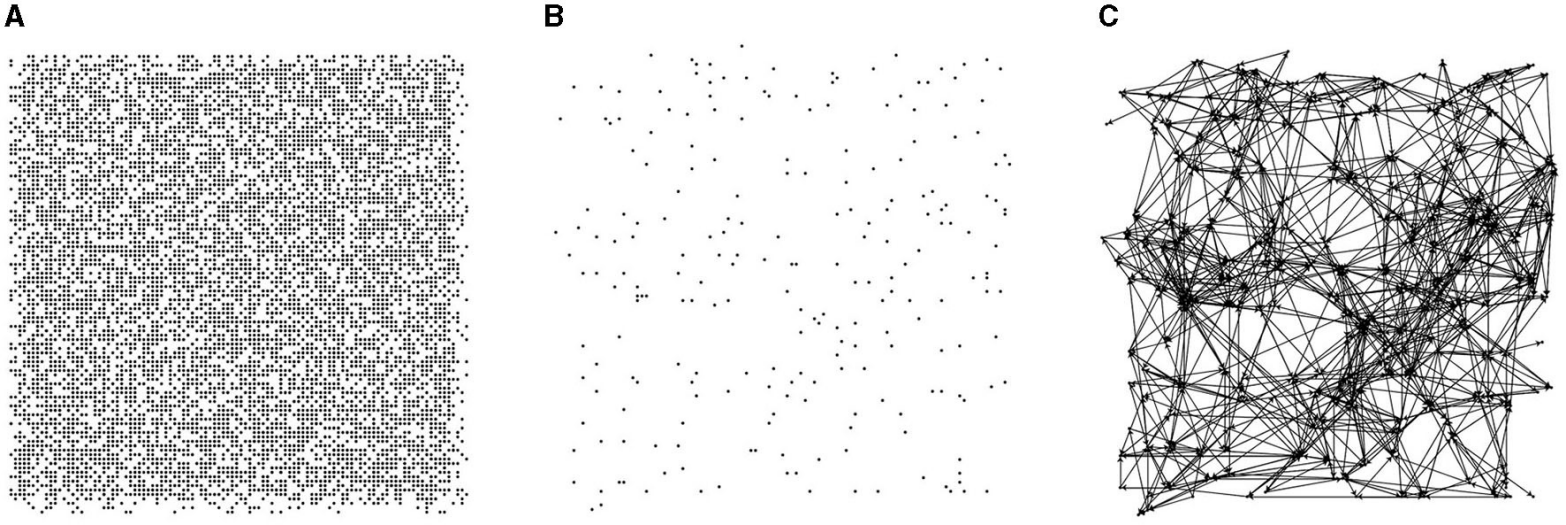
Steps of directed graph generation. **(A)** Candidate nodes. **(B)** Random node selection. **(C)** Probability-based node connections.

In this paper, 10,000 candidate nodes are generated within a 500 × 500 region, and each node's position is randomized. During directed graph generation, nodes are randomly selected from the 10,000 candidates with a specific probability (2% in this paper), and each node's contact area is randomly generated within a predetermined range. The contact area of a node is a limited resource. When establishing a directed edge, the connection probability between two nodes is related to their Euclidean distance. The connection probabilities for nodes with Euclidean distances of 0–50, 50–100, and 100–200 are 10, 20, and 5%, respectively. Figure 1 illustrates the directed graph generation process, which involves three steps: generating candidate nodes, selecting directed graph nodes, and establishing directed edges.

The primary feature of the directed graph model constructed in this paper is that the nodes form finite, random connections within a local neighborhood, and the connection probability varies depending on the distance between nodes. The pseudo-code for the directed graph generation strategy is presented in Algorithm 1.

**Algorithm 1:** Directed graph generation algorithm.

**Input:** The set of candidate nodes, *V*_*opt*_; The number of candidate nodes, *N*_*opt*_.
**Output:** The nodes of directed graph, *V*_*graph*_; The number of nodes in the directed graph, *N*_*graph*_; The adjacency matrix of directed graph, *E*; The maximum available resources for the nodes in the directed graph, *R*; The activation threshold for the nodes in the directed graph, *T* = {*t*_1_, *t*_2_, …, *t*_*N*_*graph*__}.
1: *V*_*graph*_⇐∅, *N*_*graph*_⇐0
2: **for** *i* = 1 → *N*_*opt*_ **do**
3: *B*_*i*_⇐*Node*_*Random*_*Selection*_*Judgment*()//*B*_*i*_ is a boolean value. True and false correspond to selected and unselected respectively.
4: **if** *B*_*i*_ == True **then**
5: *V*_*graph*_⇐*V*_*graph*_∪{*V*_*opt*_[*i*]}
6: *N*_*graph*_⇐*N*_*graph*_+1
7: **end if**
8: **end for**
9: *R*⇐*Random*_*Node*_*Resource*(*N*_*graph*_)//*R* records the maximum available resources of each node.
10: *T*⇐*Random*_*Node*_*Threshold*(*N*_*graph*_)//*T* records the activation threshold of each node.
11: *E*⇐*Init*_*Adjacency*_*Matrix*(*N*_*graph*_)//*E* is an adjacency matrix of size *N*_*graph*_ × *N*_*graph*_
12: **for** *i* = 1 → *N*_*graph*_ **do**
13: **for** *j* = 1 → *N*_*graph*_ **do**
14: *B*_*ij*_⇐*Node*_*Random*_*Connection*_*Judgment*(*i, j*) //*B*_*ij*_ is a boolean value. True and false correspond to connected and disconnected respectively.
15: **if** *B*_*ij*_ == True **then**
16: *E*[*i*][*j*]⇐*True*
17: **end if**
18: **end for**
19: **end for**
20: **return** *V*_*graph*_, *N*_*graph*_, *E*, *R*, *T*

### 3.3. Directed graph memory mechanism

In this paper, we represent neuronal networks as directed graph models and investigate the working mechanism of memory from the perspective of directed graphs. Nodes in a directed graph symbolize individual neurons, while directed edges denote connections between neurons.

**Figure 2:**
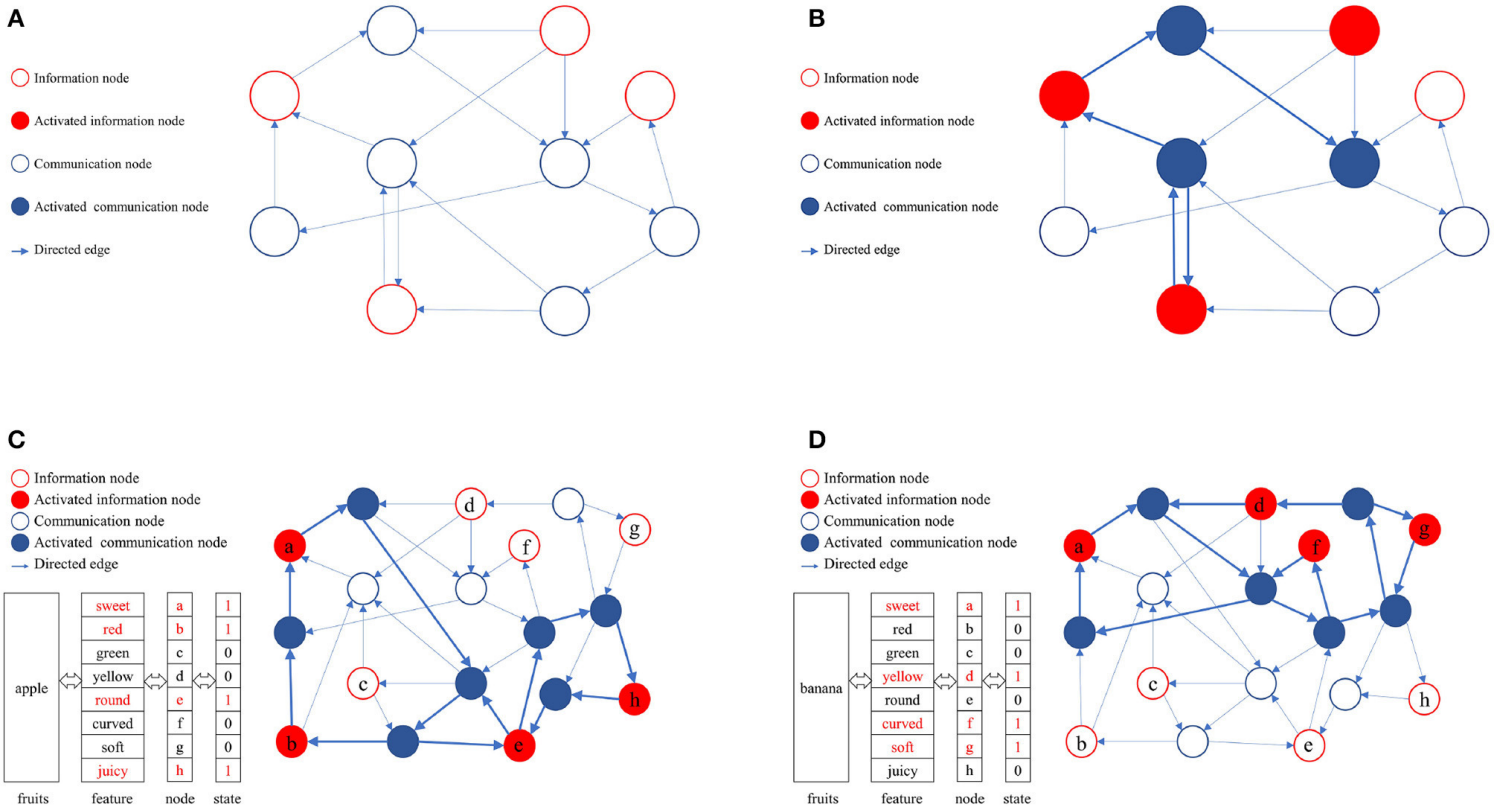
Physical mechanism of memory based on directed graph. **(A)** A blank directed graph. **(B)** A directed graph being loaded a memory. **(C)** The directed graph being loaded the memory of apple. **(D)** The directed graph being loaded the memory of banana.

In a directed graph, nodes can be categorized into information nodes and communication nodes. Information nodes serve to represent content or cluster-encode stimuli, such as the shape, size, color, taste, and other physical attributes of apples. Information nodes are responsible for the input and output of memory content, which is represented in the directed graph as an activated combination of information nodes. To maintain the activation combination of information nodes, communication nodes must establish activation paths. The connected subgraphs formed by these activation paths constitute the physical realization of memory content in the directed graph. A directed graph can encompass multiple paths, each corresponding to different memory contents. The ability to distinguish these activation paths, ensure their compatibility, minimize interference, and efficiently utilize limited node resources is attained through self-organized incremental learning of directed graphs. During the directed graph training process, communication nodes coordinate activation paths according to the directed graph node adaptive connectivity learning algorithm. The directed graph is trained to align the activation combinations of awakened information nodes as closely as possible with the initial input combinations. Figure 2 illustrates the memory implementation on a directed graph. Figure 2A displays an empty directed graph without memory content loaded, while Figure 2B presents a directed graph with memory content loaded.

In this study, the directed graph model utilizes communication nodes to connect active information nodes and form connected subgraphs based on the adaptive connectivity learning algorithm. These subgraphs physically represent the memory content within the directed graph. The stability of the memory content relies on the strength of bidirectional connectivity in the subgraphs, such as the accessibility of information nodes to one another.

As depicted in Figures 2C, D, various memory contents are stored in a directed graph, with apples and bananas corresponding to distinct activation combinations. The connected subgraphs, formed by communication nodes linking information nodes, implement these memory contents within the directed graph. During the storage and consolidation phase, it is crucial to distinguish and ensure compatibility between these subgraphs while minimizing interference, ultimately enhancing the storage capacity and quality of directed graphs. Consequently, the adaptive connectivity learning algorithm for directed graph nodes must fulfill three requirements: (1) store memory content using minimal directed graph resources, (2) differentiate connected subgraphs representing distinct memory contents, and (3) minimize interference between connected subgraphs. This serves as the foundation for designing the adaptive connectivity learning algorithm for directed graph nodes based on resource competition.

## 4. Adaptive connectivity learning algorithm for directed graph nodes

In this study, we introduce the “Node-inner Autonomous and Adaptive Connectivity Learning Algorithm Pervading in a Directed Graph Based on Resource Competition and Path Pruning” (RCPP-NCLA). Neurobiology suggests that synapses closer to the cell body or the axon hillock are used more frequently and possess a pronounced post-synaptic efficacy in both polarizing and depolarizing effects, indicating the presence of synaptic morphological plasticity (Tanaka et al., 2008; De Vincenti et al., 2019). This understanding provides two critical insights. First, the cell body's surface area, where upstream axons form contact, is a limited resource. Second, the occupation of these limited resources can undergo dynamic adjustments through competitive learning. Inspired by these insights, we devise a node-inner autonomous and adaptive connectivity learning algorithm simulating node resource competition, grounded in resource competition. We propose that the memory content is represented as an activation combination of information nodes in a directed graph, with its intrinsic physical realization being a subgraph composed of activation paths. To reduce the resource consumption of activation paths, we also suggest a resource occupation optimization strategy based on path pruning.

### 4.1. Neurobiological constraints

Biological neural networks commonly exhibit a small-world topology, characterized by numerous locally connected nodes and a relatively small number of remote connections. This topology optimizes effective communication within brain networks and reduces node connectivity costs (Stampanoni Bassi et al., 2019). In the neocortex, where local circuits play a crucial role in cortical computation, the majority of neuronal synapses originate from neighboring neurons within the same cortical region, with only a few stemming from long-distance connections (Douglas and Martin, 2007). Owing to the topology of biological neural networks, individual nodes cannot possess a global view or overarching perspective; instead, each node adjusts its behavior based on local information derived from upstream and downstream node interactions. Consequently, biological neural networks are distributed, self-organizing, and adaptive, with global-level functions achieved by nodes through local interactions.

Although great progress has been made in artificial neural networks, these models do not bear much resemblance to real neurobiological networks, and the success of artificial neural networks simply cannot be used to explain the operating mechanisms of biological neural networks. Most artificial neural network models maintain consistency in node connectivity, with the number of connections and their range remaining fixed, even though the strength of these connections can vary. These models often lack sparse behavior of nodes and exhibit low resistance to localized network damage, deviating considerably from biological reality. Current popular and successful deep learning network models contain hundreds of layers, adjustable as needed, and can display significant variation in network structure for different tasks within the same category, illustrating task specificity. Moreover, models like the Hopfield network possess a fully connected topology, allowing each node to observe the entire network's behavior and thus granting each node a global view. Yet, most artificial neural network models overlook differences in neurons' morphological and electrophysiological properties, simplifying the complex neuronal structure to a singular point. In contrast, biological neural networks are dynamic, with distinct neuronal subtypes demonstrating varied stimulus selectivity and contributing differently to cortical computation. The intricate neural dynamics are a product of individual neuronal properties and network connectivity, embodying the autonomous dynamics of biological neural networks (Sadeh and Clopath, 2021).

The specific cognitive capabilities of humans and higher mammals may hinge on distinct structural and functional aspects of neurobiological networks and require interpretation based on these neurobiological traits (Pulvermüller et al., 2021). Consequently, this paper proposes node-inner autonomous and adaptive connectivity learning algorithms for directed graph nodes, grounded in neurobiological characteristics like neurons' local field of view, autonomy, and competition for limited resources, thereby enhancing their bio-interpretability.

### 4.2. Node adaptive learning algorithm based on resource competition

In the design of the RCPP-NCLA algorithm, every node within the directed graph is allocated a fixed amount of resources. Upstream nodes utilize their access frequency as a metric for resource competition aimed at the target node. Should an upstream node exhibit a high frequency of access, it has the potential to occupy a majority of the available contact area within the target node, potentially inhibiting input from other upstream nodes.

#### 4.2.1. Access frequency

Let's assume the indegree of node *v*_*i*_ is denoted by *n*. The access frequency, $F_{i}=\left\{f_{1}^{i},f_{2}^{i},...,f_{n}^{i}\right\}$, characterizes the frequency with which upstream nodes access node *v*_*i*_. The frequency of node access inherently influences the contact area, with more frequent input leading to a larger contact area.

#### 4.2.2. Node resources

In this context, a node's resources correspond to its contactable area within the directed graph. Although the total area of each node remains constant, the percentage of the contact area occupied by each upstream node varies. This contact area is dynamically adjusted according to the access frequency. The contact area of a directed graph node *R* is the set of contact areas of each node in the directed graph, denoted as *R* = {*r*_1_, *r*_2_, …, *r*_*N*_*graph*__}, where *N*_*graph*_ represents the number of nodes in the directed graph. Using node *v*_*i*_ (with an indegree of *n*) as an example, the total contact area of node *v*_*i*_ is *r*_*i*_. The contact area allocation table $A_{i}=\left\{a_{1}^{i},a_{2}^{i},...,a_{n}^{i}\right\}$ for node *v*_*i*_ showcases the contact area occupied by each upstream node. Since the total contact area of node *v*_*i*_ is finite, *A*_*i*_ must satisfy the condition $r_{i}\geq \overset{n}{\underset{j=1}{\sum }}a_{j}^{i}$.

#### 4.2.3. Resource competition

The initial contact area for all upstream nodes is zero, and *S* represents the contact area change step. When considering node *v*_*i*_, $a_{j}^{i}$ is the value assigned to the contact area of the *j*th upstream node of node *v*_*i*_. If node *v*_*i*_ possesses an assignable free contact area, the equation $a_{j}^{i}=a_{j}^{i}+S$ is applied. However, if node *v*_*i*_ lacks an assignable contact area, it seizes resources from other nodes based on the access frequency.

**Figure 3:**
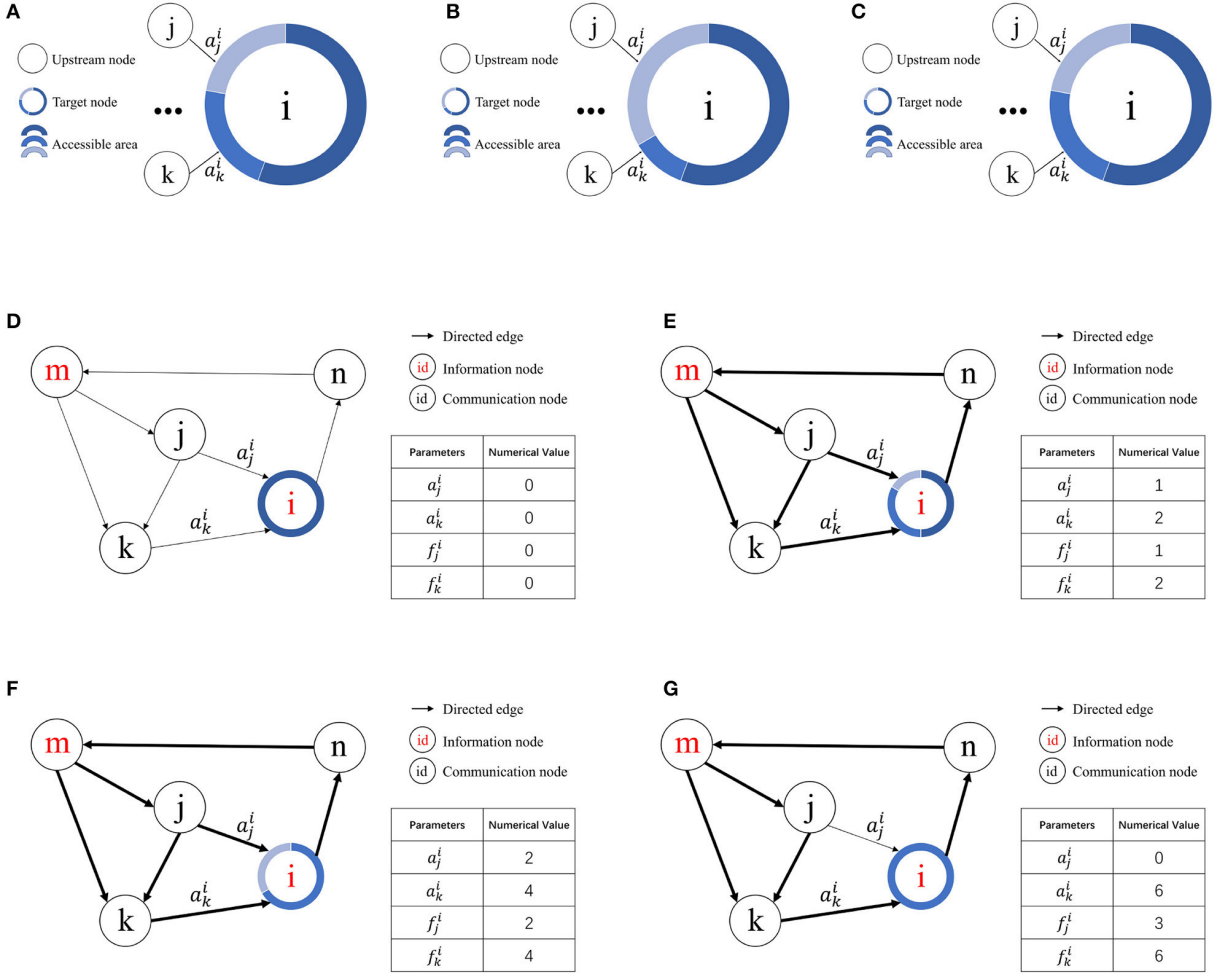
Illustration of resource competition among nodes. **(A)** Resource allocation in node *v*_*i*_ prior to access by node *v*_*j*_. **(B)** Resource acquisition by node *v*_*j*_ from node *v*_*k*_ for node *v*_*i*_ given $f_{j}^{i}\geq f_{k}^{i}$. **(C)** Unchanged resource allocation in node *v*_*i*_ when $f_{j}^{i}<f_{k}^{i}$. **(D)** Initial state. **(E)** Node *i* allocates contact area to node *j* and node *k* for the first time. **(F)** Node *i* allocates contact area to node *j* and node *k* for the second time. **(G)** Node *k* wins the resources obtained by node *j*.

According to the definition of access frequency, $f_{j}^{i}$ represents the access frequency of the *j*th upstream node of node *v*_*i*_. Suppose the *k*th upstream node of node *v*_*i*_ exhibits the lowest access frequency among nodes with a contact area greater than zero. If $f_{j}^{i}\geq f_{k}^{i}$, then $a_{j}^{i}=a_{j}^{i}+S$ and $a_{k}^{i}=a_{a}^{i}-S$. If no node *v*_*k*_ satisfies these conditions, the contact area $a_{j}^{i}$ of node *v*_*j*_ remains unchanged. A schematic of resource competition is shown in Figures 3A–C.

Figures 3D–G illustrates how the RCPP-NCLA directed graph model learns the activation path corresponding to a memory sample by adjusting the contact area. The aim of the proposed learning algorithm is to learn activation paths with limited resources. In Figure 3E, node *v*_*i*_ allocates the available free contact area to node *v*_*j*_ and node *v*_*k*_. Since there are two paths through node *v*_*k*_ to reach node *v*_*i*_, both the contact area $a_{k}^{i}$ and the access frequency $f_{k}^{i}$ of node *v*_*k*_ are equal to 2. In Figure 3F, node *v*_*i*_ has allocated all the available free contact area. In Figure 3G, because node *v*_*k*_ has a higher access frequency, it wins the contact area that was previously assigned to node *v*_*j*_.

The resource competition algorithm serves to dynamically allocate the contact area of targeted nodes. When the target node contains unallocated contact areas, they can be directly occupied by upstream nodes. However, if all contact areas within the target node are already allocated, upstream nodes must compete for these areas based on access frequency. In this scenario, upstream nodes with a lower access frequency are more likely to lose their occupied contact area to those with higher frequencies. The pseudocode for this resource competition algorithm is presented in Algorithm 2.

**Algorithm 2:** Resource competition algorithm.

**Input:** Index of the accessing upstream node, *V*_*a*_; Maximum available resources of the target node, *r*_*target*_; Number of upstream nodes connected to the target node, *Indegree*; Access frequency to the target node, *F*_*target*_; The resource allocation table of target node,$A_{target}=\left\{a_{1}^{target},a_{2}^{target},...,a_{Indegree}^{target}\right\}$; The step of resource allocation, *S*.
**Output:** The number of visits to target node after update, *F*_*target*_; The resource allocation table of target node after update, *A*_*target*_;
1: $f_{V_{a}}^{target}⇐f_{V_{a}}^{target}+1$//Increment *V*_*a*_ node's access frequency to the target node.
2: **if** $S+\overset{Indegree}{\underset{i=1}{\sum }}a_{i}^{target}\leq r_{target}$ **then**
3: $a_{V_{a}}^{target}⇐a_{V_{a}}^{target}+S$
4: **else**
5: *V*_*b*_⇐*Comparison*_*Visit*(*F*_*target*_, *A*_*target*_, *V*_*a*_)//*V*_*b*_ denotes the node that possesses the target node's resources and has a lower access frequency than *V*_*a*_.
6: **if** *V*_*b*_ > 0 **then**//Check if the node index is valid.
7: $a_{V_{a}}^{target}⇐a_{V_{a}}^{target}+S$
8: $a_{V_{b}}^{target}⇐a_{V_{b}}^{target}-S$
9: **end if**
10: **end if**
11: **return** *F*_*target*_, *A*_*target*_

**Algorithm 3:** RCPP-NCLA.

**Input:** The number of directed graph nodes, *N*_*graph*_; The number of initial activated nodes, *N*_*in*_; Initial activated nodes, $V_{in}=\left\{v_{1}^{in},v_{2}^{in},...,v_{N_{in}}^{in}\right\}$; The adjacency matrix of directed graph, *E* = (_*e*_*ij*_)*N*_*graph*_×*N*_*graph*__; The maximum available resources of directed graph nodes, *R* = {*r*_1_, *r*_2_, …, *r*_*N*_*graph*__}; The activation threshold of directed graph nodes, *T* = {*t*_1_, *t*_2_, …, *t*_*N*_*graph*__}; The access times table of directed graph node, *F* = (_*f*_*ij*_)*N*_*graph*_×*N*_*graph*__; The resource allocation table of directed graph nodes, *A* = (_*a*_*ij*_)*N*_*graph*_×*N*_*graph*__; The matrix of path preference, *P* = (_*p*_*ij*_)*N*_*graph*_×*N*_*graph*__.
**Output:** Output node set,*V*_*out*_.
1: *C*⇐*Init*_*Node*_*Charge*()//The cumulative charge of directed graph nodes, *C* = {*c*_1_, *c*_2_, …, *c*_*N*_*graph*__}.
2: *Heap*⇐*Init*_*Heap*()//The heap to manage the access order of nodes according to the timestamp.
3: *Time*⇐*Current*_*Time*()//Current timestamp.
4: *S*⇐*Set*_*Step*()//S is the step of resource allocation. The default value of S is 1.
5: *V*_*out*_⇐∅// Initializes the output node set.
6: **for** $i=v_{1}^{in}\to v_{N_{in}}^{in}$ **do**
7: *Target*_*Set*⇐*Get*_*All*_*Outdegree*_*Node*(*E, i*)
8: *Heap*⇐*Heap*_*Manager*_*Add*(*Heap, Target*_*Set, Head, E*)//The heap manager calculates the timestamp and adds the target node to the heap.
9: **end for**
10: **while** *Heap*_*Is*_*Not*_*Empty*(*Heap*) **do**
11: *Head, Tail*⇐*Heap*_*Pop*(*Heap*)//The heap pops up the head and tail of next arc. The head is the target node to be accessed.
12: *Indegree*⇐*Get*_*Indegree*(*E, Head*)
13: *F*[*Head*], *A*[*Head*]⇐*Resource*_*Competition*_*Algorithm*(*Tail*, *R*[*Head*], *Indegree, F*[*Head*], *A*[*Head*], *S*)
14: *C*[*Head*]⇐*C*[*Head*]+*A*[*Head*][*Tail*]//The cumulative charge of target node.
15: **if** *C*[*Head*]≥*T*[*Head*] **then**
16: **if** *Is*_*Info*_*Node*(*Head*) and *Is*_*Not*_*In*_*Set*(*V*_*out*_, *Head*) **then**
17: *V*_*out*_⇐*V*_*out*_∪{*Head*}//Add the activated information node to *V*_*out*_.
18: *P*⇐*Update*_*Path*_*Preference*(*P, Tail, Head, E*)//In the subsequent training, the path that can reach the information node is preferred.
19: **else**
20: if *Have*_*Preference*_*Node*(*P, Head*) **then**
21: *Target*_*Set*⇐*Get*_*Preference*_*Node*(*P, Head*)//Node Head preferentially activates the node that can reach the information node.
22: **else**
23: *Target*_*Set*⇐*Get*_*All*_*Outdegree*_*Node*(*E, Head*)
24: **end if**
25: *Heap*⇐*Heap*_*Manager*_*Add*(*Heap, Target*_*Set, Head, E*)
26: **end if**
27: *C*[*Head*]⇐0
28: **end if**
29: **end while**
30: **return** *V*_*out*_

### 4.3. Resource occupancy optimization strategy based on path pruning

The path pruning module in RCPP-NCLA functions to minimize the resource consumption of activation paths by pruning those unable to reach an activated information node. This process not only conserves the limited resources of nodes and paths but also enhances the stability and recall of the activation paths.

Let's consider *V* as the set of nodes in the directed graph, represented as *V* = {*v*_1_, *v*_2_, …, *v*_*n*_}, where *n* denotes the total number of nodes. This set includes both information nodes and communication nodes. The set of information nodes, *M*, is defined as $M=\left\{v_{1}^{m},v_{2}^{m},...,v_{s}^{m}\right\}$, with $v_{i}^{m}$ representing the *i*-th information node and *s* indicating the quantity of information nodes. These information nodes can be in two states: active (1) or inactive (0), and the initial active state corresponds to the memory content.

**Figure 4:**
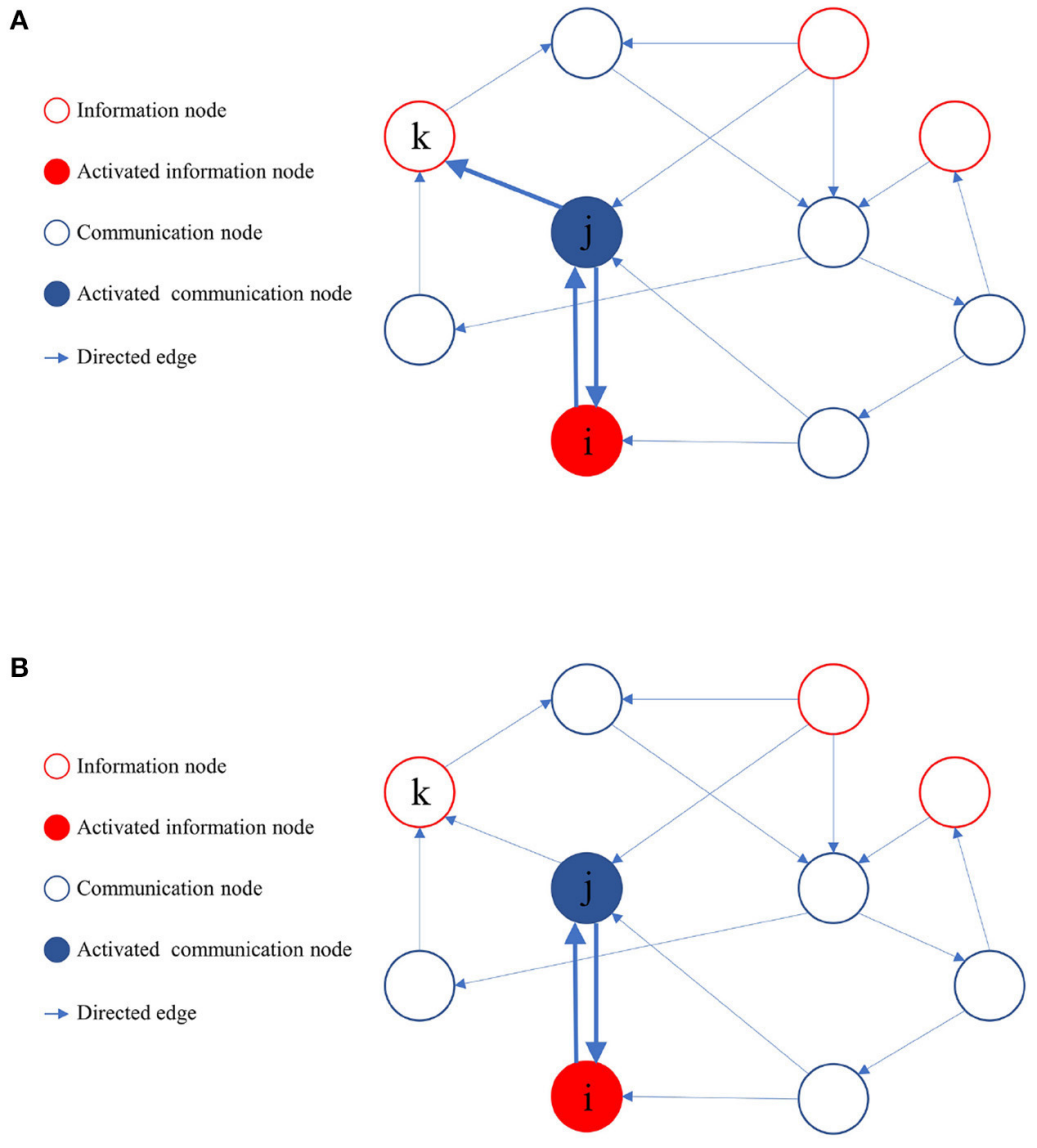
Illustration of path pruning. **(A)** Update of path preference upon access to an inactive information node *v*_*k*_ by the communication node *v*_*j*_. **(B)** During subsequent training, the communication node *v*_*j*_ avoids accessing the information node *v*_*k*_.

During the learning process of the directed graph, if a node *v*_*j*_ visits an information node $v_{i}^{m}$ from the set *M*, and the initial activation state of $v_{i}^{m}$ is active, it implies that a path from *v*_*j*_ to the information node in the active state exists. Node *v*_*j*_ then provides feedback to all its upstream nodes and laterally inhibits other sibling nodes that cannot reach the activated information nodes. The upstream nodes of node *v*_*j*_ also reciprocate feedback to their respective upstream nodes until it reaches the source of the activation path. As the learning process proceeds, when node *v*_*j*_ activates a downstream node, only those downstream nodes capable of reaching the activated information node get activated. Figure 4 illustrates the process of path pruning.

RCPP-NCLA mitigates the consumption of node and path resources related to memory samples by pruning invalid activation paths, thereby enhancing the recall accuracy of memory samples. Algorithm 2 outlines the pseudocode of RCPP-NCLA.

### 4.4. Resource occupancy optimization strategy based on lateral inhibition

In this work, we also develop a resource occupation optimization strategy that draws inspiration from the concept of lateral inhibition in neurobiology. Lateral inhibition, a process in which neuronal cells form synergy directly or indirectly, plays a crucial role in controlling neuronal firing and shaping the circuit's output (Fan et al., 2020). Motivated by this mechanism, our strategy minimizes the number of activation paths via lateral inhibition among sibling nodes, thereby diminishing the size of the subgraphs corresponding to memory samples. This approach aims to mitigate interference among memory samples and augment their recall accuracy.

## 5. Experimental design and performance analysis

In this section, we have designed an experimental approach based on cognitive behavior to test the memory performance of the active directed graph model proposed in this study. The experiments in this section encompass directed graph testing, activation path testing, performance testing of the learning algorithm, ablation studies, and comparative experiments. These experiments are conducted with a focus on three aspects: the basic properties of directed graphs, the performance of adaptive learning algorithms for directed graph nodes, and the performance comparison of associative memory models.

### 5.1. Basic properties of directed graphs

**i. Experimental objectives:** Test of basic properties of directed graphs.

In this paper, we propose a directed graph generation strategy in Section 3.2, which can batch create a number of larger and statistically similar directed graphs for performance testing. In this section, we evaluate the directed graphs generated by this strategy for their reachability, clustering coefficient, and average path length.

**ii. Experimental methods**

The directed graph's node scale spans from 10 to 500, with an increment of 10 units between scales, resulting in a total of 50 sets of test samples. Each set comprises 10 directed graphs with identical node scales but varying topologies, and the experimental outcomes are computed as the average across each set of test samples. Let us denote the directed graph as *G* = < *V, E*>, where *V* = {*v*_1_, *v*_2_, …, *v*_*n*_}, and *n* represents the count of nodes in the directed graph.

In graph theory, reachability refers to the ease of moving from one node to another within a graph. In this study, we devised the average percentage of reachable nodes as a metric for assessing the reachability of directed graphs. (1) RM (Reachability Matrix): The reachability matrix of the directed graph, denoted as *RM* = (*rm*_*ij*_)*n*×*n*, where *rm*_*ij*_ is 1 or 0, indicating whether *v*_*i*_ to *v*_*j*_ is reachable or not, respectively. (2) NRPM (Number of Reachable Paths Matrix): The number of reachable paths matrix is represented as *NRPM* = *RM*+(*RM*)^2^+(*RM*)^3^+…+(*RM*)^*n*−1^. Based on NRPM, we can determine the count of paths between two nodes with a path length not exceeding *n*−1. (3) APRN (Average Percentage of Reachable Nodes): The proportion of reachable nodes to the total nodes in the directed graph is derived from NRPM. We employ the mean value of APRN as an evaluative index to measure the reachability of directed graphs in this study.

The clustering coefficient, an evaluation metric used in graph theory, depicts the degree of graph clustering. Clustering coefficients can be categorized into global and local coefficients. The global clustering coefficient assesses the overall graph's clustering degree, defined as the ratio of closed triple point groups to the connected triple point groups within the graph. The local clustering coefficient evaluates the clustering degree of a node in relation to its neighboring nodes. For instance, for node *v*_*i*_, it is defined as the ratio of the actual number of directed edges present between the neighboring nodes of node *v*_*i*_ to the potential maximum number of directed edges. In this study, the average value of local clustering coefficients of directed graph nodes is used as an evaluation index to measure the degree of graph aggregation.

The average path length is the mean value of the path lengths between nodes in the directed graph, with the path length representing the shortest distance between nodes.

**iii. Experimental results**

**Figure 5:**
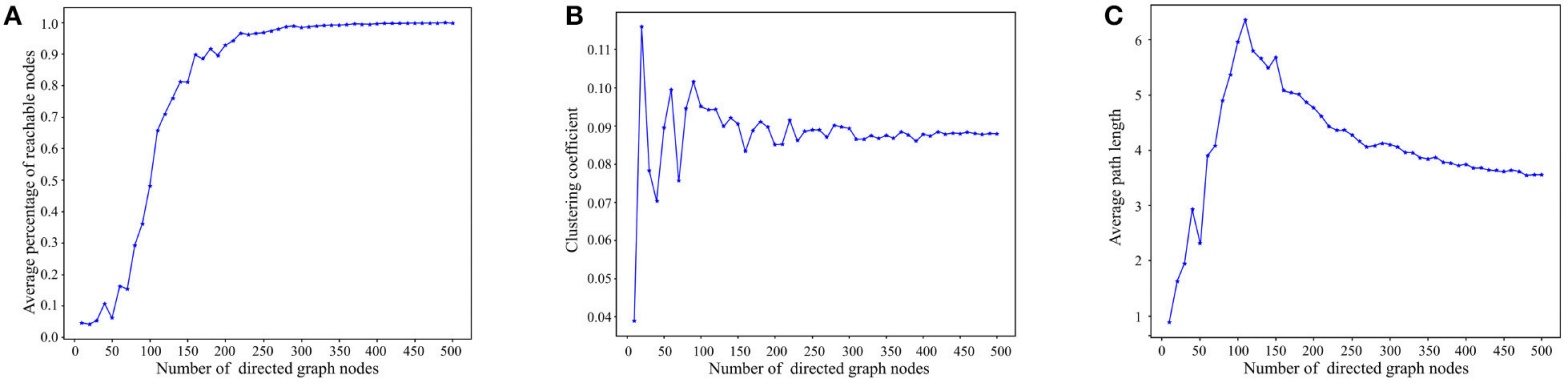
Experimental results on basic properties of directed graphs. **(A)** Reachability statistics of directed graphs with different node scales. **(B)** Clustering coefficient statistics of directed graphs with different node scales. **(C)** Average path length statistics of directed graphs with different node scales.

Figure 5A presents the reachability statistics for directed graphs of varying node scales. The experimental findings indicate that the Average Percentage of Reachable Nodes (APRN) escalates with the node scale and remains above 90% when the node scale exceeds 200. Consequently, we select directed graphs with a node scale of 200 for the subsequent tests.

Figure 5B depicts the outcomes of the directed graph clustering coefficient tests across various node scales. At smaller node scales, the nodes within the directed graph are disjointed. However, with the increment of node scales, the clustering coefficient progressively stabilizes at ~8–9%. This indicates that the degree of clustering among the nodes in the directed graph eventually reaches stability.

Figure 5C presents the results of the average path length tests across diverse node scales. As depicted in Figure 5B, the average path length tends to increase with node scale until the node scale reaches 110. As node size enlarges, reachable paths between nodes gradually form, primarily through short-range edges, leading to a progressive increase in the average path length. However, when the node scale surpasses 110, the nodes in the directed graph become denser, and the average path length starts to decline due to the increasing presence of long-range edges in the path between nodes, ultimately stabilizing between 3 and 4. In consideration of Figures 5A, C, in directed graphs with substantial node scales, long-range edges significantly reduce the path length between nodes while preserving high reachability.

### 5.2. Activation path experiments

This subsection conducts activation path experiments on RCPP-NCLA, designing both iterative training for a singular memory sample and incremental training for multiple memory samples. These experiments aim to assess the stability rate and recall rate of the RCPP-NCLA activation path. Furthermore, this subsection provides visual representations of the memory samples within a directed graph.

#### 5.2.1. Iterative training of a single memory sample

**i. Experimental objectives:** Evaluating single-sample iterative training performance in RCPP-NCLA directed graph model.

This subsection evaluates the stability of the directed graph model's activation path, focusing on resource consumption, time cost, and memory stability as key indices. The physical manifestation of memory samples within directed graphs takes the form of connected subgraphs. In this context, the experiment calculates the number of directed edges associated with the connected subgraph for each memory sample. The average of memory samples of the same scale serves as the resource consumption index for the directed graph. Time cost is assessed through the number of training iterations, indicating the count of iterations required for memory samples to form stable connected subgraphs within the directed graphs. Memory stability, gauged through the activation path stability rate, elucidates the stability of the connected subgraph associated with the memory samples. Additionally, this experiment offers a visual demonstration of a single memory sample's iterative training.

**ii. Experimental methods**

**Figure 6:**
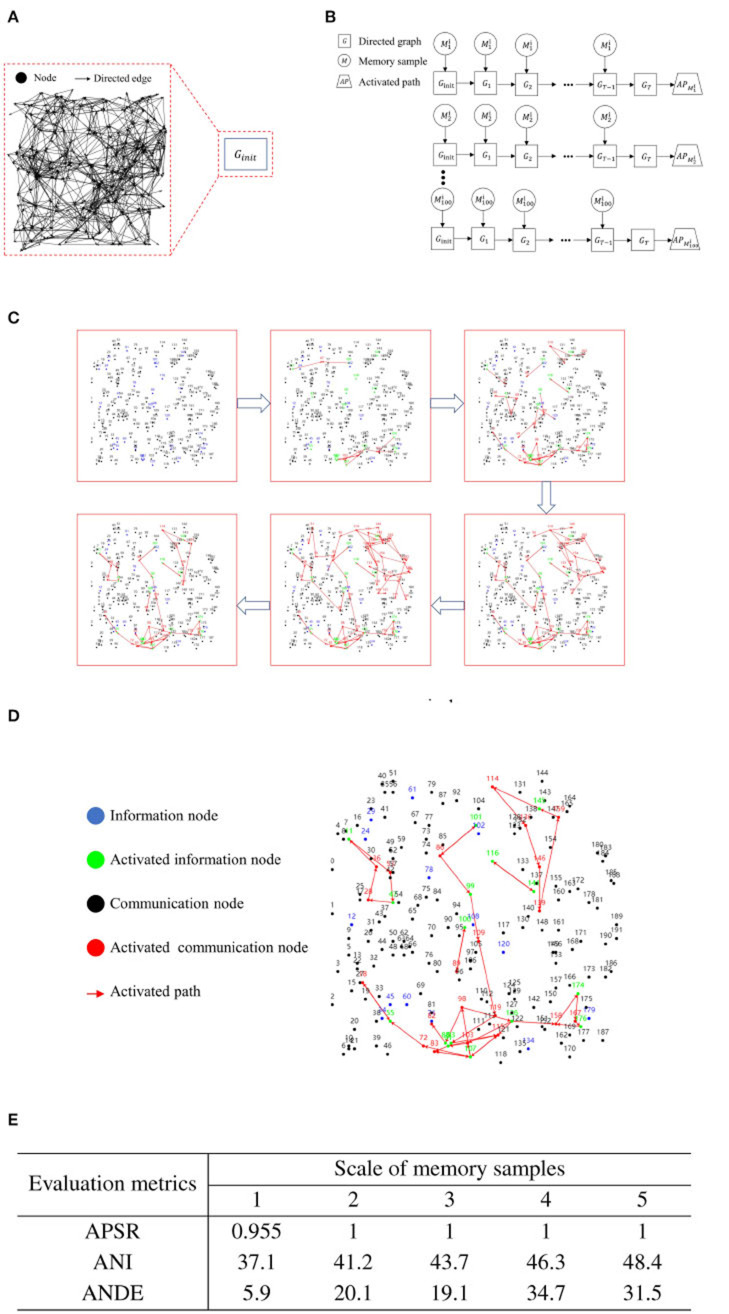
Experimental results for iterative training of a single memory sample. **(A)** Initial state of a blank directed graph. **(B)** Memory sample uploading and process of directed graph state transformation. **(C)** Activation path iteration diagram for a single memory sample. **(D)** Example diagram of recall test after iterative training of a single memory sample. **(E)** Activation path stability of RCPP-NCLA directed graph model.

In the same directed graph, for each case, we generate 100 memory samples with scales of 1, 2, 3, 4, and 5 nodes randomly. We denote the memory sample as $M_{j}^{i}$, where *i* represents the memory sample scale, and *j* signifies the ordinal number among the memory samples of the same scale. In the initial state of the directed graph, we apply iterative training to a single memory sample, capping the number of iterations at 100 and setting the maximum depth for each iteration to 10. For clarity, we define the directed graph obtained at the t-th iteration as *G*_*t*_. The iterative training halts if the activation path stabilizes. If there is no change in the activation path and the node contact area over 20 iterations, we consider the activation path as stable. We define the activation path corresponding to the memory sample $M_{j}^{i}$ as $AP_{M_{j}^{i}}$. Figures 6A, B presents the flowchart of iterative training for a single memory sample.

**iii. Experimental results**

Figure 6C illustrates the progressive stabilization of the activation path during the iterative training of a singular memory sample. Initially, and in the mid-stages of training, communication nodes aim to activate as many neighboring nodes as possible to learn the routes leading to activated information nodes. As training progresses to the latter stages, the routes reaching the activated information nodes are continually reinforced until the activation paths achieve a state of stability.

Figure 6D illustrates the activation path associated with the recall test. As demonstrated in Figure 6D, the directed graph node adaptive connectivity learning algorithm optimizes the combination of activated information nodes to closely mirror the initially deposited combination.

**Figure 7:**
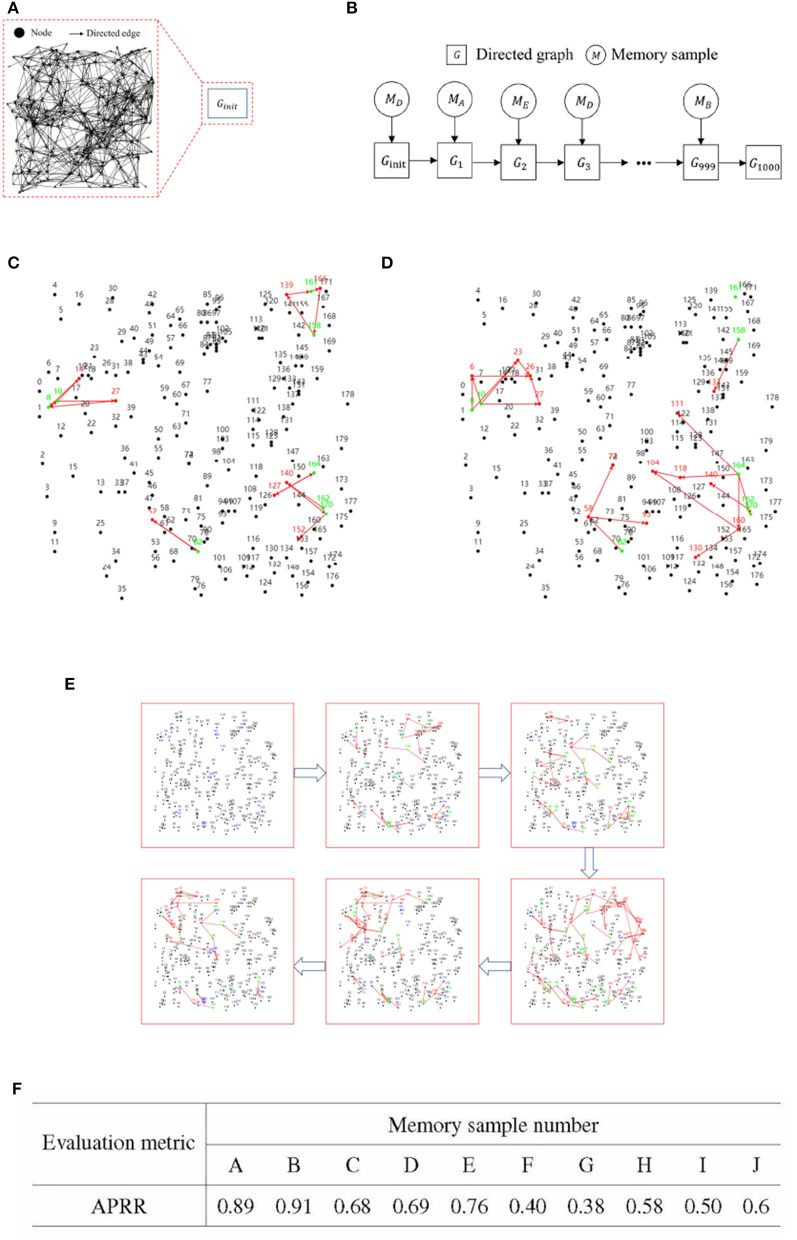
Experimental results for incremental training of multiple memory samples. **(A)** An initial blank directed graph. **(B)** Memory sample uploading and directed graph state change process.**(C)** Effective memory retrieval. **(D)** Incomplete memory retrieval. **(E)** Iteration diagram for incremental training of multiple memory samples. **(F)** Recall rate of activation path of RCPP-NCLA directed graph model.

Figure 7E presents the experimental outcomes from the iterative training of a single memory sample. This table provides three evaluation metrics: Activation Path Stability Rate (APSR), Average Number of Iterations (ANI), and Average Number of Directed Edges (ANDE). APSR represents the proportion of memory samples that can achieve stable activation paths, ANI denotes the number of iterations required for the memory samples to attain stability in activation paths, and ANDE indicates the quantity of directed edges utilized by the activation path corresponding to the memory samples. As demonstrated in Figure 6E, the RCPP-NCLA directed graph model yields stable activation paths, with APSR reaching 95.5% for the dataset with a memory sample scale of 1 and achieving 100% for all other memory sample scales.

#### 5.2.2. Incremental training of multiple memory samples

**i. Experimental objectives:** Assessing Incremental Training Memory Performance in RCPP-NCLA directed graph model.

This subsection evaluates the incremental storage performance of the RCPP-NCLA directed graph model. Additionally, it provides a visual depiction of the incremental training process involving multiple memory samples.

**ii. Experimental methods**

All experiments were conducted on the same directed graph. Ten instances of memory samples, each with different scales and all capable of achieving activation path stabilization, were selected for incremental training. Here, each memory sample is denoted as *M*_*i*_, where *i* stands for the number of the memory sample. In this study, 100 sets of incremental training were executed on an initial state directed graph. For each incremental training set, a total of 1,000 rounds of memory training were performed. The memory samples for each round were randomly selected, with each round having a maximum depth of 10. Each of the 1,000 memory trainings was based on the current state of the directed graph, allowing for the subsequent memory training. The directed graph obtained after the t-th round of training is defined as *G*_*t*_.

Upon completion of 1,000 sessions in each set of incremental training, recall tests were conducted on each of the ten memory samples. The primary metric for these recall tests was the Activation Path Recall Rate (APRR). Let the stable activation path obtained from iterative training of a single memory sample be denoted as *E*_*T*_, and *E*_*T*_ is defined as the positive class. The activation path obtained from incremental training of multiple memory samples is defined as *E*_*p*_. FN represents the unactivated correct path. TP refers to the path that is correctly activated within *E*_*p*_. Figures 7A, B presents the flowchart for the incremental training of multiple memory samples.


(1) $$\begin{array}{l} APRR=\frac{TP}{TP+FN} \end{array}$$

Figures 7C, D gives a demonstration of activation paths, Figure 7C shows valid activation paths where information nodes are correctly activated and Figure 7D shows incomplete activation paths where some of the information nodes are not activated and the activation paths take up too much of the directed graph resources.

**iii. Experimental results**

Figure 7E illustrates the process of incremental training with multiple memory samples within a directed graph model. This method involves randomly selecting different memory samples for inclusion in the directed graph. Compared to the iterative training of a single memory sample, the incremental training of multiple memory samples introduces competition and interference between memory samples. The recall accuracy of memory samples is directly related to the stability and connectivity of their corresponding subgraphs. Figure 7F presents the results of incremental training for ten memory samples, demonstrating that the RCPP-NCLA directed graph model can achieve a high activation path recall rate and can effectively retain most of the activation paths corresponding to each memory sample.

### 5.3. Performance experiments

In this section, we will evaluate the directed graph preference and capacity of the RCPP-NCLA directed graph model. The experimental details are outlined in Table 1 to provide a comprehensive overview of the conducted experiments.

**Table 1:** Introduction to the performance experiments.

**Experiments**	**Objectives**	**Methods**	**Evaluation metrics**
Training sample preference experiments	Analyzing the preference of the directed graph by training it with 1,000 memory samples and evaluating the recall test results.	Incremental training of multiple memory samples	Information node status recall accuracy
Directed graph topology preference experiments	Analyzing the preferences of different directed graphs by training them with 1,000 memory samples and evaluating the recall test results.	Incremental training of multiple memory samples	Information node status recall accuracy
Directed graph storage capacity experiments	Observing the storage capacity of the directed graphs by training them with memory samples of varying sizes.	Incremental training of multiple memory samples	Information node status recall accuracy

#### 5.3.1. Training sample preference experiments

**i. Experimental objectives:** Evaluating the preference of directed graph models toward training samples.

The experiment involved the random generation of 1,000 memory samples for incremental training. Subsequently, the recall test was conducted on these 1,000 memory samples. The recall test results were used to analyze and validate the memory samples preferred and not preferred by the directed graph model. The objectives of this experiment are twofold: (1) To test the potential preferences of the directed graph model based on the recall test results after incremental training. (2) To design subsequent positive and negative examples based on the initial test results.

**ii. Experimental methods**

In this experiment, 1,000 memory samples were randomly generated for incremental training with a memory sample dimension of 30. The evaluation index is IRA (Information Node State Recall Accuracy), which is the correct rate of information node state output by the directed graph. Information nodes have both active (1) and inactive (0) states. Information nodes are used to characterize content, i.e., they are responsible for the input and output of memory content. Memory content is represented in the directed graph as the activation combinations of information nodes. Let the number of information nodes be *N*_*info*_, and the number of information nodes with correct activation status in the output information node activation combinations is *N*_*true*_.


(2) $$\begin{array}{l} IRA=\frac{N_{true}}{N_{info}} \end{array}$$

The directed graph model underwent incremental training, with each set of incremental training performed on the initially defined directed graph. Within each set, a total of 10,000 memory training iterations were conducted. The memory samples for each training iteration were randomly selected, with a maximum depth of 10. Throughout the 10,000 training sessions, each subsequent session was based on the current state of the directed graph. This experiment examines the recall accuracy of the directed graph model after incremental training.

**iii. Experimental results**

After the 1,000 memory samples underwent recall testing, they were screened based on their recall accuracy. In this experiment, two sets of memory samples were selected based on the recall accuracy threshold. The first set included memory samples with a recall accuracy of 65% or higher, which were defined as memory samples preferred by the directed graph. Additionally, memory samples with a recall accuracy below 45% were included in the second set and considered as memory samples not preferred by the directed graph model.

**Figure 8:**
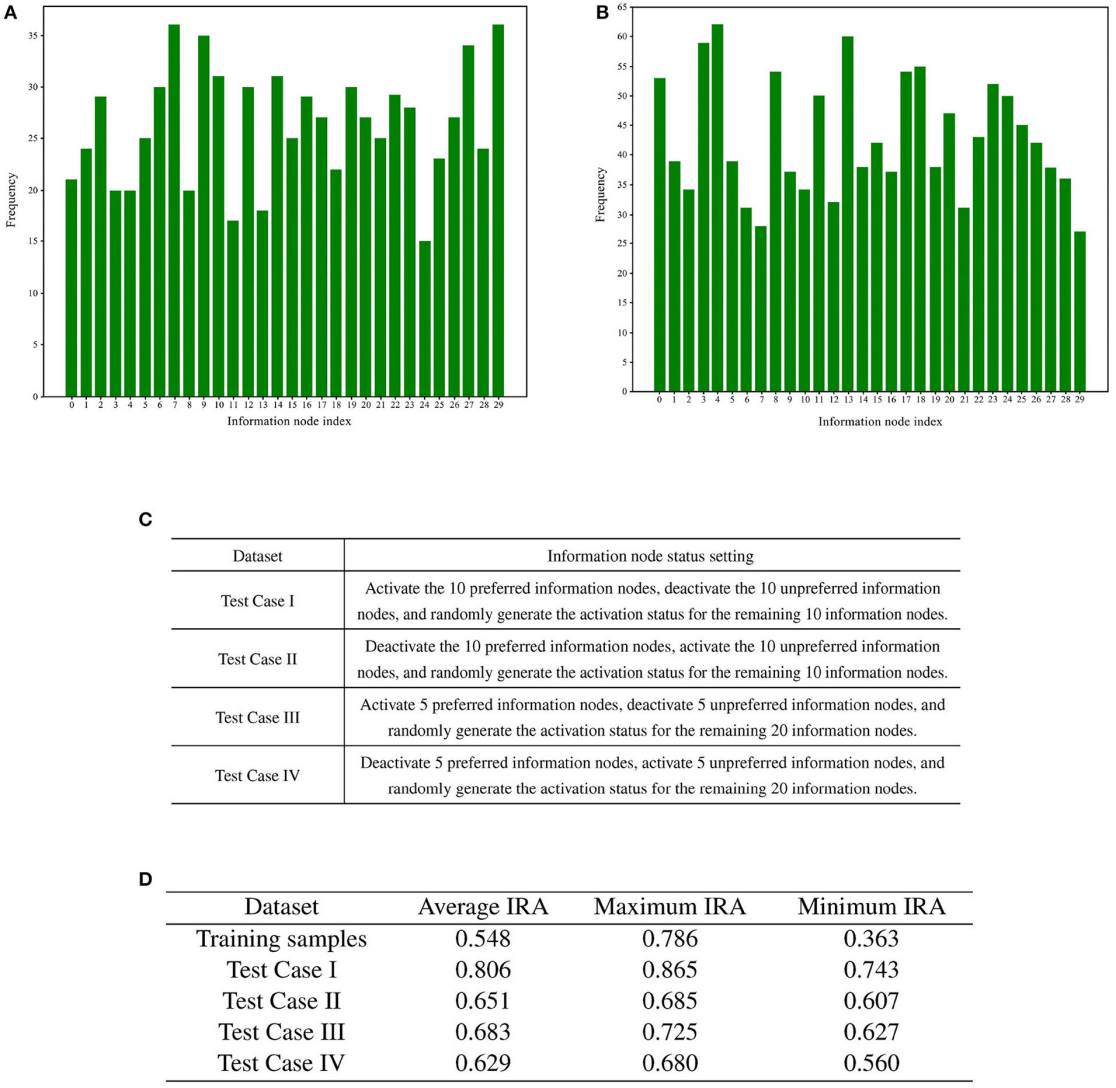
Preference test for training memory samples. **(A)** Bar chart illustrating the number of activations for each information node in memory samples with high recall accuracy. **(B)** Bar chart illustrating the number of activations for each information node in memory samples with low recall accuracy. **(C)** Rules for generating test cases. **(D)** Results of training sample preference experiments.

Following the screening process, the two sets of memory samples were subjected to analysis. The cumulative number of activations of each information node in these two sets of memory samples was calculated. A total of 30 information nodes were assigned numbers from 0 to 29. The statistical analysis of the cumulative number of activations for the information nodes is presented in Figures 8A, B.

To statistically examine the hypothesis of directed graph preference derived from Figures 8A, B, we designed four groups of test cases for recall accuracy testing, each comprising 1,000 test cases. The generation rules for the test cases are detailed in Figure 8C.

A total of four different test cases were generated based on Figure 8C. For each of the four test cases, 100 sets of incremental training and recall tests were conducted. The experimental results are presented in Figure 8D.

The recall test results for all four test cases outperformed the recall test results when training with 1,000 randomly generated memory samples. The memory samples in the test cases exhibit similarities, leading to shared resources within the directed graph. As a result, the competition for resources among these memory samples is smaller compared to the competition among the 1,000 randomly generated memory samples. A comparison between test case one and test case three reveals that activating more information nodes preferred by the directed graph can enhance memory sample recall accuracy. Likewise, comparing Test Case I with Test Case II demonstrates that information nodes in an activation state contrary to the directed graph preference can lower the recall accuracy of the memory samples.

This paper introduces connected subgraphs as mechanisms for representing memory content in directed graphs. The node adaptive learning algorithm maps the memory contents to connected subgraphs in the directed graph. Through training sample preference experiments, we demonstrate the existence of preferences for training samples in directed graph models. Specific combinations of activated information nodes can significantly improve or impair the model's performance compared to the average. This phenomenon occurs because the experimental results depend on the correspondence between input information and previously learned connected subgraphs. For instance, if the directed graph model has learned about apples with features like red and circular, inputting features related to red and circular facilitates the association between apples and the directed graph model, and vice versa. Furthermore, the resource competition module enables continuous learning capability within the directed graph model. If the initial learning suggests that apples are blue but subsequent extensive training indicates that apples are red, the model will adjust and re-associate apples with the color red.

#### 5.3.2. Directed graph topology preference experiments

**i. Experimental objectives:** Investigating the Topology Preference of Directed Graph Models.

This experiment involved incremental training on different directed graphs and analyzed their preferences based on recall test results. The objective was to examine the relationship between variations in directed graph topology connections and their preferences, and to determine if the overall memory performance of the directed graph model remains unaffected by the graph's topology.

**Figure 9:**
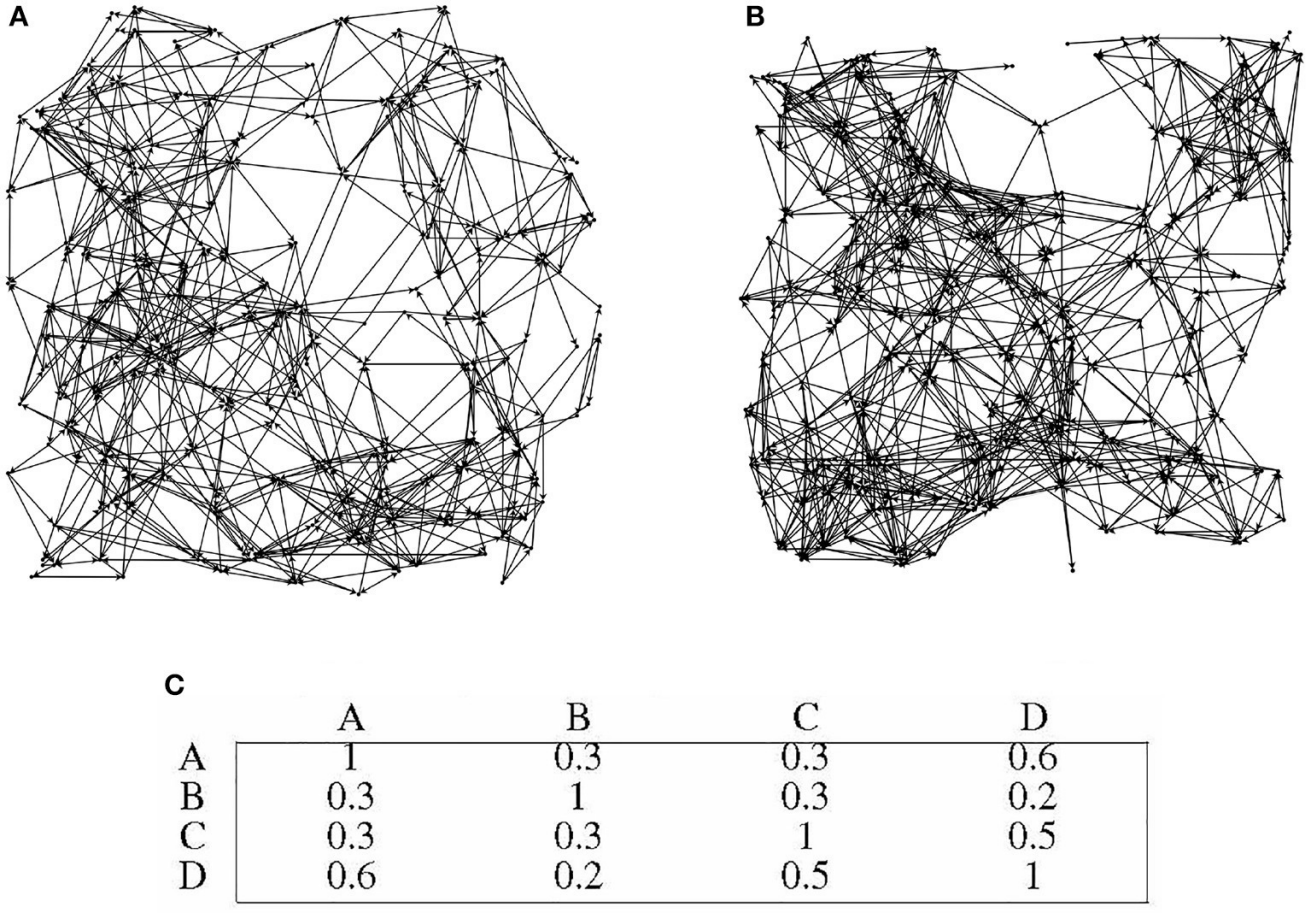
Differences in topological structures among different directed graphs. **(A)** directed graph A. **(B)** directed graph B. **(C)** Preference similarity statistics of different directed graphs.

Four distinct directed graphs, labeled as A, B, C, and D, were utilized for this experiment. In order to illustrate the disparities between the graphs, Figures 9A, B depicts the topology of graphs A and B.

**ii. Experimental methods**

In this experiment, the memory sample dimension was set to 30, and the evaluation index used was the IRA (Information Node State Recall Accuracy). Among the four directed graphs, 30 nodes were designated as information nodes with identical numbering, while the remaining nodes served as communication nodes. The numbering of these 30 information nodes remained consistent across the different directed graphs, while the topological connections of each directed graph were generated randomly.

For the purpose of incremental training, 1,000 memory samples were generated randomly, and all four directed graphs were trained using the same set of 1,000 memory samples. Each directed graph underwent incremental training individually. Following the completion of incremental training, recall tests were conducted on the 1,000 memory samples. The results of the recall tests were then used to analyze the preferences of each directed graph toward the memory samples.

**iii. Experimental results**

Figure 9C presents the similarity statistics for the preferences of the four directed graphs. It is worth noting that the 30 information nodes in each directed graph are uniformly numbered, indicating that the preference of each directed graph is closely tied to its unique topology. Importantly, the overall performance of the directed graph model remains unaffected by the specific topology of the graph. The directed graph's node adaptive algorithm learns distinct connected subgraphs based on the topology of each specific directed graph.

#### 5.3.3. Directed graph storage capacity experiments

In this subsection, we conduct information node correlation analysis and capacity experiments to evaluate the storage performance of the directed graph model. Connected subgraphs play a crucial role in constructing the graph structure for storing information in the directed graph model. The information node correlation analysis enables us to statistically identify pairs of information nodes that are more likely to occur together. Additionally, the capacity experiments are designed to assess the storage capacity of directed graph models across datasets of varying scales.

**A. Information node correlation analysis**

**i. Experimental objectives:** Analyzing the relevance of information nodes.

A significant number of training samples exhibit specific combinations of features, and the directed graph model has the ability to capture these frequent combinations and form connected subgraphs. These subgraphs serve as fundamental components for storing information in the directed graph model. Information nodes that consistently occur together are more likely to be part of the same connected subgraph, which aligns with Hebb's law.

The objective of this experiment is to investigate the correlation among information nodes. The directed graphs are trained using datasets of varying sizes, and the occurrence frequency of different information nodes within the same subgraph is recorded.

**ii. Experimental methods**

The memory samples in this experiment have a dimension of 15, and their scale ranges from 1 to 1,000. The evaluation index used is INC (Information Node Correlation). By conducting recall tests, we can extract the connected subgraphs associated with the memory samples. The frequency at which information nodes appear together within the same connected subgraph is defined as the information node correlation. *INC* = (*inc*_*ij*_)*n*×*n*, *inc*_*ij*_ denotes the frequency of node *v*_*i*_ and node *v*_*j*_ appearing in the same subgraph.

**iii. Experimental results**

**Figure 10:**
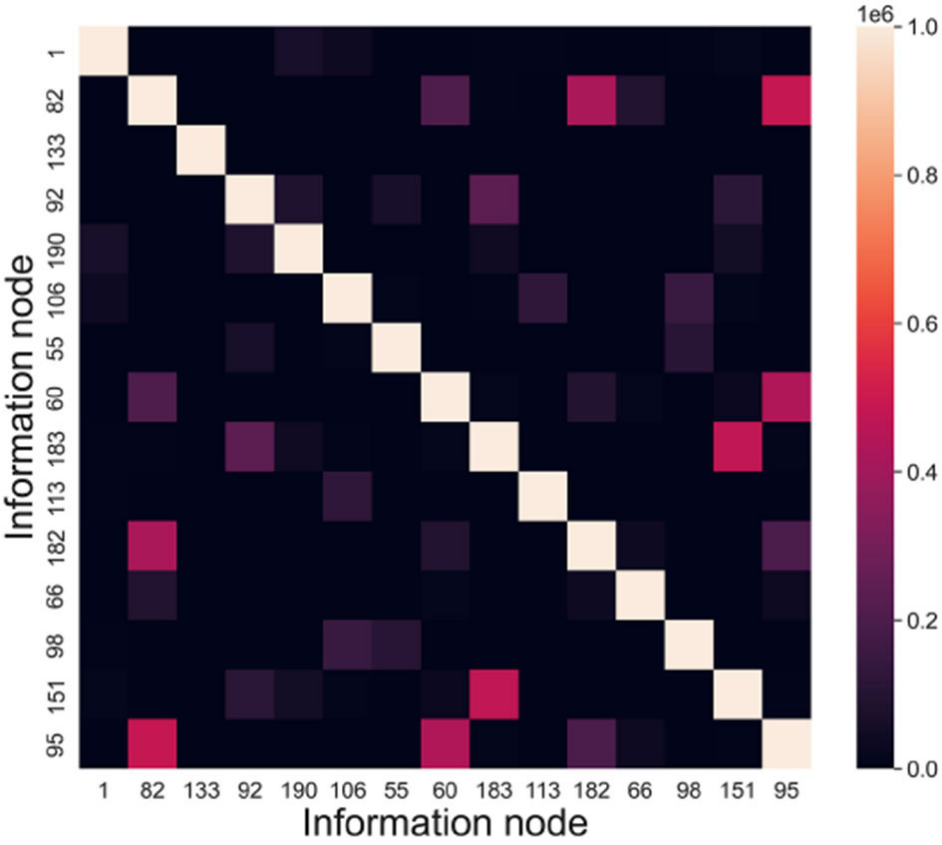
Information node relevance statistics chart.

According to the above experiments, we can get the connected subgraphs corresponding to each memory sample in the directed graph, analyze whether the information nodes appear in the same connected subgraph and count the frequency. Taking node *v*_*i*_ and node *v*_*j*_ as an example, in a recall test, if these two nodes are in the same connected subgraph, then *inc*_*ij*_ = *inc*_*ij*_ + 1, and *inc*_*ji*_ = *inc*_*ij*_. Figure 10 presents the information node relevance statistics chart.

**B. RCPP-NCLA directed graph model storage capacity experiments**

**i. Experimental objectives:** Testing the storage capacity of the RCPP-NCLA directed graph model.

The objective of this experiment is to conduct capacity tests on the RCPP-NCLA directed graph model and evaluate the recall accuracy of the model across different memory sample scales and dimensions.

**ii. Experimental methods**

The memory samples used in this experiment have a dimension of 15, indicating that there are 15 information nodes, and the evaluation index used is IRA (Information Node State Recall Accuracy). We conducted two memory capacity tests. The first test involved memory samples of different scales, ranging from 300 to 32,700 with 300-unit intervals. The second test compared the memory performance of the proposed model for different memory sample dimensions. The memory sample dimensions are 15 and 20. The memory sample scales range from 50 to 1,000 with an interval of 10.

**iii. Experimental results**

**Figure 11:**
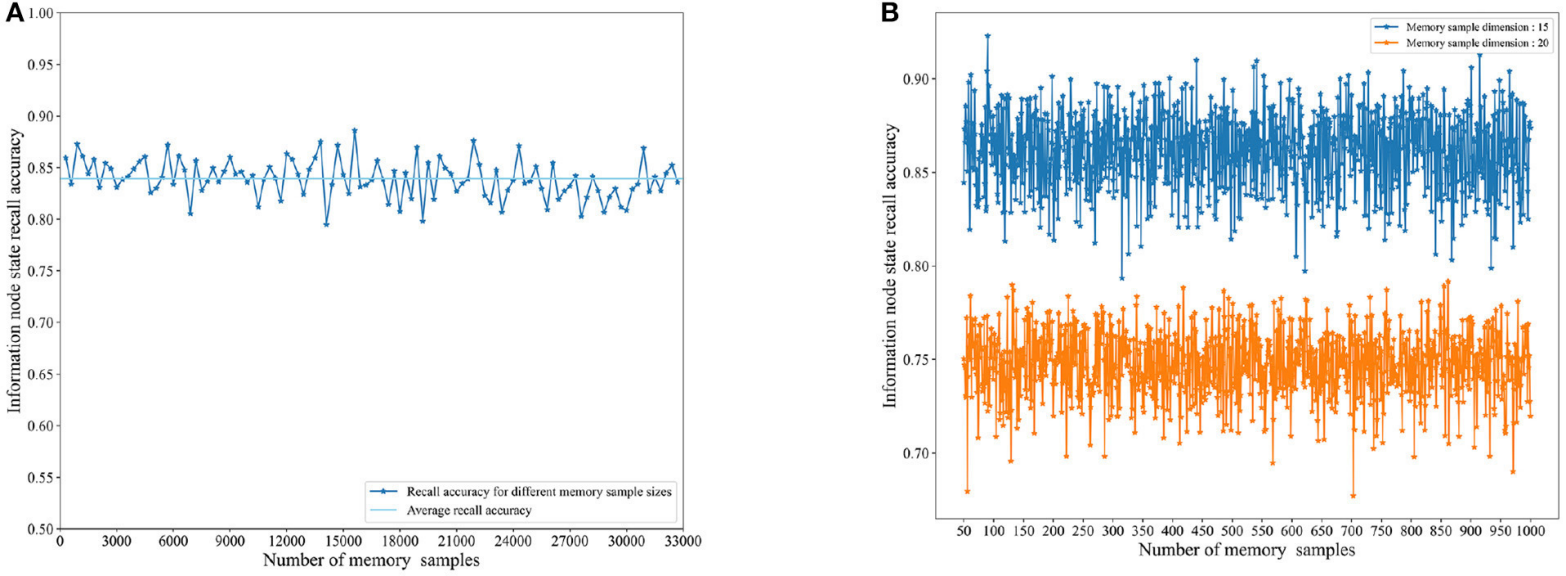
Memory capacity test statistical chart. **(A)** Capacity testing of different memory sample scales. **(B)** Capacity testing of different memory sample dimensions.

Figure 11 illustrates the recall accuracy for various memory sample sizes and dimensions. In Figure 11A, the RCPP-NCLA directed graph model is capable of maintaining a recall accuracy at or above 80% as the memory sample size increases. This experimental result is closely related to the resource competition module. Connected subgraphs represent the physical realization of memory samples in a directed graph; a memory sample contains one or more connected subgraphs, and those that occur more frequently are more likely to be remembered. In Figure 11B, an increase in the dimension of memory samples impacts the recall accuracy of the RCPP-NCLA directed graph model. A higher memory sample dimension implies that a single memory sample needs to utilize more directed graph resources, consequently leading to increased interference among memory samples.

### 5.4. Ablation experiments

**i. Experimental objectives:** Evaluating the impact of the path pruning module on memory performance.

The PP (Path Pruning) module enhances memory performance by reducing the resource consumption of activation paths. In this subsection, the memory performance of three directed graph node adaptive learning algorithms, namely RC-NCLA, RCLI-NCLA, and RCPP-NCLA, is compared. RC-NCLA incorporates the RC (Resource Competition) module, RCLI-NCLA incorporates both the RC (Resource Competition) module and the LI (Lateral Inhibition) module, while RCPP-NCLA includes the RC (Resource Competition) module and the PP (Path Pruning) module.

The directed graph models based on these three learning algorithms are trained using iterative and incremental methods. The recall test results provide insights into the enhancement of memory performance achieved by the PP module.

**ii. Experimental methods**

The three nodal adaptive learning algorithms underwent iterative training using a single memory sample and incremental training using multiple memory samples on directed graphs with identical topology and dataset. For the iterative training of a single memory sample, the dataset consisted of 100 memory samples with scales ranging from 1 to 5. The evaluation indices used were APSR (Activation Path Stability Rate), ANI (Average Number of Iterations), and ANDE (Average Number of Directed Edges). In the case of incremental training with multiple memory samples, the dataset included ten memory samples with scales between 1 and 5, and the evaluation index used was APRR (Activation Path Recall Rate).

**iii. Experimental results**

**Figure 12:**
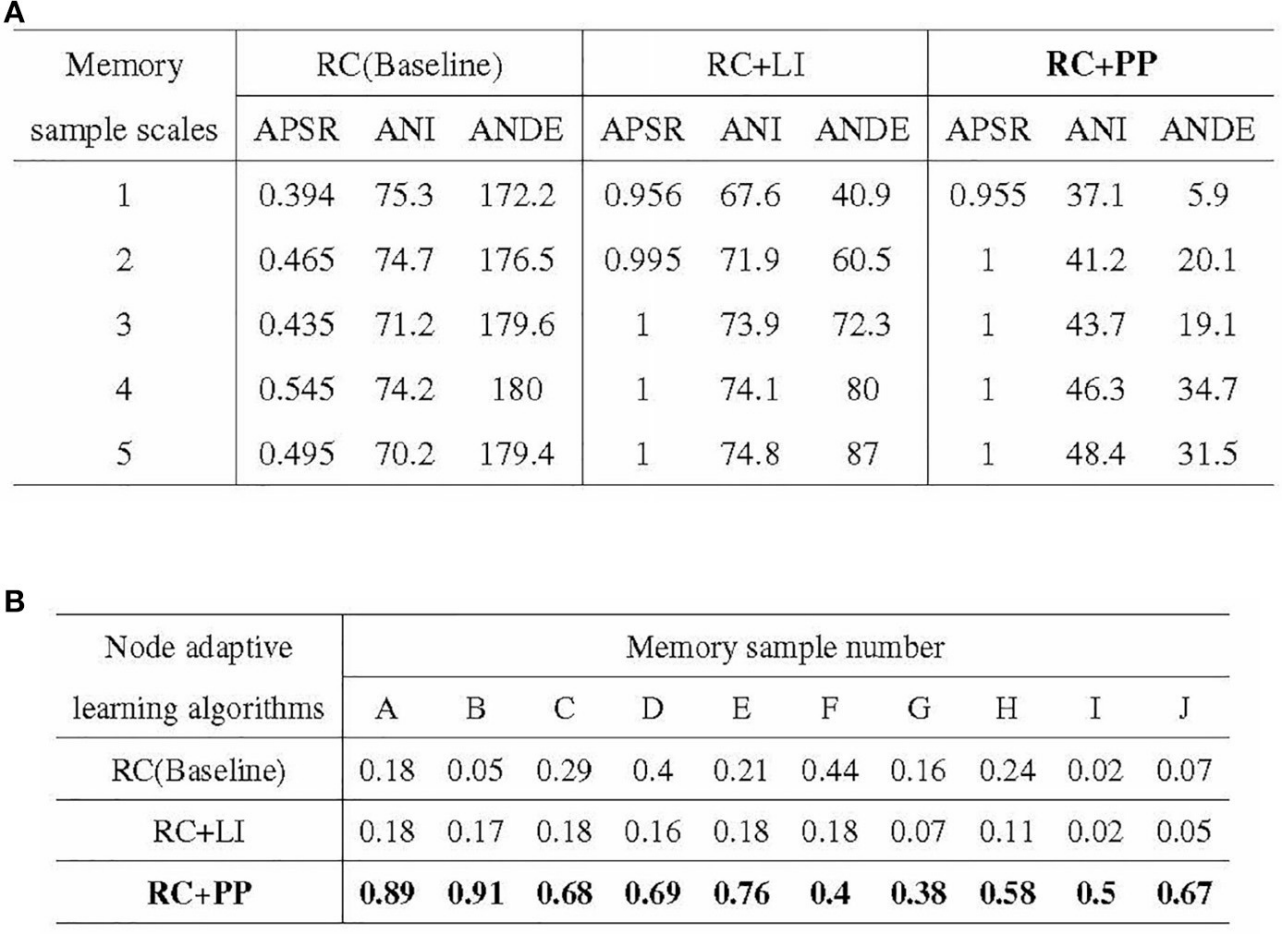
Results of ablation experiments. **(A)** Stability of activation paths in different directed graph models. **(B)** Recall rate of activation path in different directed graphs models.

The experimental results of the ablation experiments are presented in Figure 12. Figure 12A illustrates the outcomes of the iterative training with a single memory sample, while Figure 12B showcases the results of the incremental training with multiple memory samples. In Figure 12A, it can be observed that the PP module significantly reduces the resource consumption of directed graph memory samples. The ANDE for memory samples with scales 1–5 was 3.4, 11.4, 10.6, 19.3, and 17.6% of the Baseline, respectively. Additionally, the PP module effectively reduces the time cost, as reflected by the lowest ANI among the three nodal adaptive learning algorithms. The LI module, another optimization strategy proposed in this study to reduce resource occupation, also decreases the resource and time overhead of memory samples. However, its optimization effect is inferior to that of the PP module. Figure 12B demonstrates the significant improvement in APRR achieved by the PP module. Although the LI module can reduce the resource and time overhead of memory samples, the APRR for most memory samples is even lower than that of the Baseline.

In conclusion, the PP module reduces the resource and time overhead of directed graph memory samples, enhances the memory stability of the directed graph model, and significantly improves the recall accuracy of memory samples.

### 5.5. Comparative experiments

#### 5.5.1. Memory performance comparison

**i. Experimental objectives:** Memory performance of RCPP-NCLA directed graph model compared with Hopfield network.

To better elucidate the memory performance of the active directed graph model proposed in this paper, a comparison is made between the RCPP-NCLA directed graph model and the Hopfield network.

**ii. Experimental methods**

Two datasets were created for this experiment, both with a memory sample dimension of 15. The dataset scales were set at 10 and 30. The RCPP-NCLA directed graph model employed an incremental training approach using multiple memory samples to learn the datasets. On the other hand, the Hopfield network determined its network weights based on the Hebb rule and employed an asynchronous approach to neuron updates. Subsequently, both the RCPP-NCLA directed graph model and the Hopfield network were evaluated for memory performance using the same datasets. The evaluation metrics used were RC (Recall Accuracy) and SC (Storage Capacity). Each sample is a binary vector of 10 or 30 neurons, and RA is the recall accuracy of the neuron state. For the RCPP-NCLA directed graph model, the formula for RA is the same as IRA. In this study, the FT (Fault Tolerance) was set to 0.85. When RA 0.85, the corresponding memory samples are regarded as valid memories, and SC reflects the number of samples that the model can effectively memorize, i.e., the number of memory samples with RA 0.85.

**iii. Experimental results**

**Figure 13:**
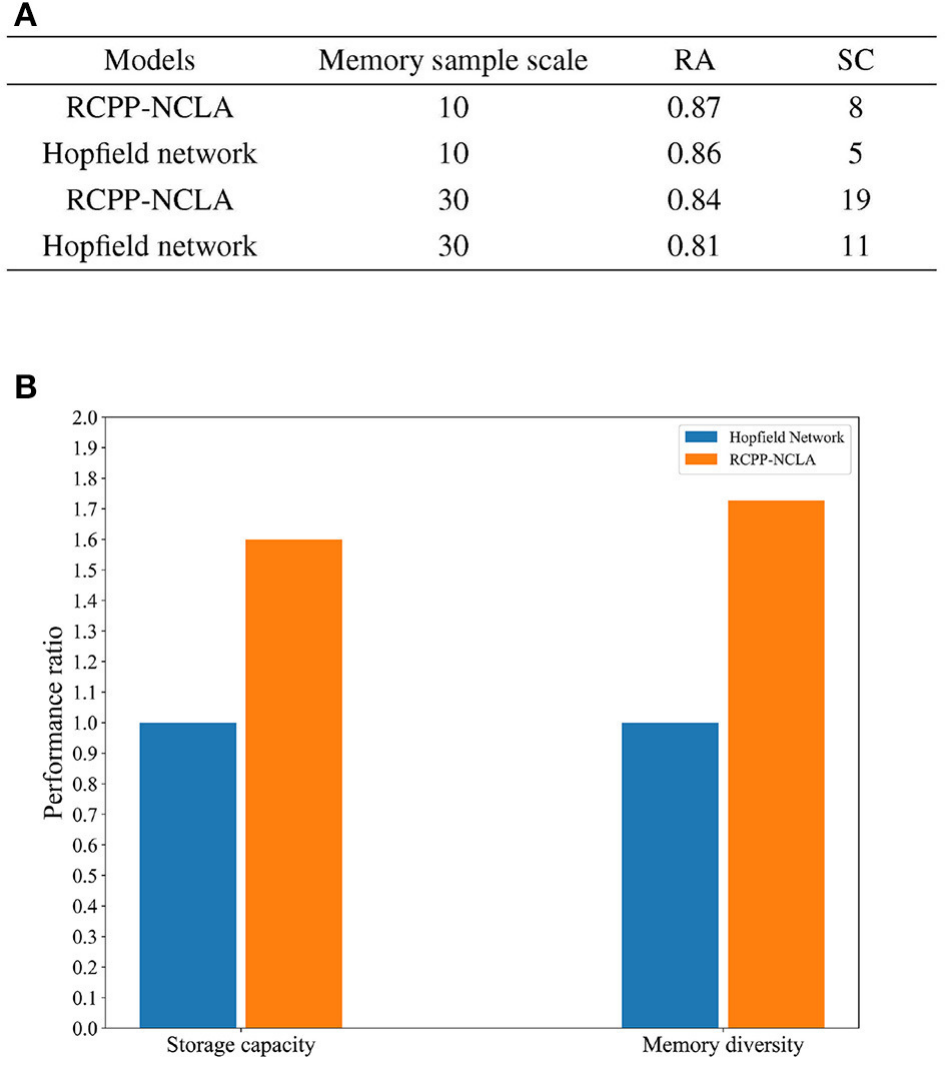
Memory performance comparison. **(A)** Results of memory performance comparison experiments. **(B)** Comparison chart of memory capacity and memory diversity.

The results of the comparative experiments between the RCPP-NCLA directed graph model and the Hopfield network are presented in Figure 13A. As shown in Figure 13A, the RCPP-NCLA directed graph model exhibited higher RA on both datasets compared to the Hopfield network, and its SC (Storage Capacity) was significantly superior.

Memory performance is not only reflected in the number of memory samples stored, but the diversity of memory samples is equally essential. Figure 13B displays the ratio of the RCPP-NCLA directed graph model to the Hopfield network in terms of memory capacity and diversity. The Hopfield network is set as the baseline with a value of 1, while the RCPP-NCLA directed graph model's value represents the ratio to the Hopfield network. Figure 13B illustrates that the RCPP-NCLA directed graph model not only achieves higher RA and SC than the Hopfield network but also exhibits a greater capacity for retaining diverse and varied memory samples.

In addition to surpassing the Hopfield network in memory performance, the RCPP-NCLA model offers additional advantages: (1) Incremental storage capability: Unlike the Hopfield network, which requires presetting weights and lacks incremental storage, the RCPP-NCLA directed graph model enables continuous learning and incremental storage. (2) Adaptability to memory samples of various dimensions: The RCPP-NCLA directed graph model can learn memory samples of different dimensions without modifying the directed graph's topology, while the Hopfield network is limited to learning memory samples of the same dimension.

#### 5.5.2. Comprehensive comparison of associative memory models

In this section, the RCPP-NCLA directed graph model is comprehensively compared with various types of associative memory models, and the results of the comparison are shown in Table 2.

**Table 2:** Comparison of associative memory models.

**Models**	**Network structure**		**Learning algorithm**		**Memory functions**	
	**Sparse connection**	**Local perspective**	**Hebb's rule**	**Adaptive learning**	**Associative memory**	**Incremental memory**
HNN (Hopfield, 1982)	×	×	✓	×	✓	×
QHAM (Miller and Mukhopadhyay, 2021)	×	×	✓	×	✓	×
BAM (Kosko, 1988)	×	×	✓	×	✓	×
MBAM (Li et al., 2021)	×	×	✓	×	✓	×
SWHNN (Sun et al., 2022)	✓	×	✓	×	✓	×
KVMN (Tyulmankov et al., 2021)	×	×	×	×	✓	✓
Ours	✓	✓	✓	✓	✓	✓

This section compared three aspects: network structure, learning algorithm, and memory function. In terms of network structure, biological cerebral cortex neural networks are characterized by local connections; spatially similar neurons are more likely to form connections, and neurons do not have a global perspective. They can only acquire information through upstream and downstream nodes. Constructing a network structure in line with neurobiology helps understand the information processing mechanism of the brain, so sparse connections and a local perspective are chosen as evaluation metrics in terms of network structure. In terms of learning algorithm, Hebb's law is a classical synaptic learning model. If the presynaptic neuron and the post-synaptic neuron are in the same state at the same moment, the synaptic connection is strengthened; if both of them are in the opposite state at the same moment, the synaptic connection is weakened. Biological neurons themselves are complex computational units with autonomous dynamics, capable of adaptive learning based on local information. Therefore, in this section, Hebb's law and adaptive learning are used as evaluation metrics in terms of learning algorithms. In terms of memory function, this section selects associative memory and incremental memory as evaluation metrics, respectively. This study focuses on associative memory, where the associative memory model is able to recall the corresponding memory content based on the input cues, and incremental memory reflects the ability of the associative memory model to learn continuously.

In Table 2, QHAM (Quantum Hopfield Associative Memory) and MBAM (Memristor Bidirectional Associative Memory) are improved models of the Hopfield network (HNN) and Bidirectional Associative Memory (BAM) network, respectively. In terms of network structure, both the fully connected features of the Hopfield network and the fully connected features of the interlayer in the BAM network make these types of memory computation models inconsistent with neurobiological constraints, such as sparse connections and local horizons. Additionally, the Hopfield network and BAM network require presetting network weights according to the learning algorithm, preventing them from realizing incremental memory. SWHNN (Small-World Hopfield Neural Network) utilizes the small-world network as the topology of the Hopfield network, which is still essentially an improved model of the Hopfield network. Although the model conforms to the characteristics of sparse connectivity, the neurons in the network have a global perspective and cannot realize incremental memory. KVMN (Key-Value Memory Network) is a key-value-based associative memory model capable of achieving the memory performance of the Hopfield network and incremental memory. However, its network structure does not align with the connectivity characteristics of the biological cortical neural network and lacks a neurobiological basis.

## 6. Conclusion

Based on neurobiology, this paper simulates the connection characteristics of biological cerebral cortex neural networks to construct directed graphs. It designs a node adaptive learning algorithm under the premise of limited resource competition, combining graph theory, multi-agent systems, PDP (Parallel Distributed Processing), ubiquitous learning, and other theories. The objective is to study the storage capacity of active directed graphs from the perspective of graph theory.

First, this paper constructs directed graphs based on biological neural networks and proposes a memory mechanism rooted in graph theory. It considers connected subgraphs as the physical realization of memory content in directed graphs. The topological structure of the directed graphs is designed based on the connection characteristics of biological cerebral cortex neural networks. The key feature is that the nodes of the directed graphs form limited and random connections within local neighborhoods, and the connection probability varies with the distance between nodes. In the RCPP-NCLA directed graph model, nodes are categorized into information nodes and communication nodes. Information nodes represent content and are responsible for the input and output of memory content. The memory content is expressed as the active combination of information nodes in the directed graphs. To maintain the activation combination of information nodes, it is necessary to establish activation paths through communication nodes. The connected subgraphs formed by these activation paths serve as the physical realization of memory content in the directed graphs. In contrast to the Hopfield network, BAM network, and their improved models, the RCPP-NCLA directed graph model offers greater biointerpretability in terms of connection mode. These network models possess a global perspective and fail to conform to neurobiological constraints.

Secondly, drawing from the neural characteristics of neurons such as local vision, autonomous initiative, and limited resource competition, this paper introduces an adaptive connected learning algorithm for nodes in directed graphs. The algorithm incorporates resource competition and path pruning. It is worth noting that many artificial neural networks (ANNs) overlook the morphological and electrophysiological differences among neurons, simplifying their complex structure into a single point. In contrast, the node adaptive connected learning algorithm implemented in directed graphs enables each node to operate independently, learn autonomously, adjust based on local information, and manifest global memory behavior through local interactions.

Finally, this paper employs experimental methods that leverage cognitive behavior to evaluate the memory performance of the RCPP-NCLA directed graph model. The experiments conducted on the RCPP-NCLA directed graph model are summarized as follows:(1) The test of basic properties of directed graphs evaluates the directed graph model from three aspects: reachability, clustering coefficient and average path length, and proves that the directed graph model designed in this paper conforms to the connection characteristics of biological cerebral cortex neural network; (2) Activation path experiments show the physical realization of memory content in directed graph, and prove that directed graph model can realize incremental storage; (3) Training sample preference experiments show that RCPP-NCLA directed graph model has preference for memory content, which is similar to brain network, and different people have different memory ability for different things; (4) The directed graph topology preference experiments reflect that the macroscopic memory performance of RCPP-NCLA directed graph model is not affected by the topological structure of directed graph, which is also similar to brain network. Everyone's brain network has differences, but its basic memory performance is basically the same; (5) In the directed graph storage capacity experiments, different data sets are designed to test the influence of sample size on the memory performance of RCPP-NCLA directed graph model. Experiments show that RCPP-NCLA directed graph model can maintain high memory accuracy under different data sets; (6) The ablation experiments prove that the path pruning module can optimize the occupation of directed graph resources by memory content, and significantly improve the stability rate and recall rate of active path; (7) In the comparative experiments, the RCPP-NCLA directed graph model is superior to Hopfield network in memory accuracy, memory capacity and memory sample diversity.

## Data availability statement

The original contributions presented in the study are included in the article/supplementary material, further inquiries can be directed to the corresponding author.

## Author contributions

HW: Visualization, Writing—original draft, Conceptualization, Methodology, Project administration, Supervision, Validation, Writing—review and editing, Formal analysis, Funding acquisition, Investigation, Resources, Software. FL: Methodology, Writing—review and editing, Data curation, Formal analysis, Investigation, Software, Visualization, Writing—original draft.
